# The NewroBus platform: engineered humanized anti-TfR1 nanobodies for efficient brain delivery

**DOI:** 10.1186/s12964-025-02605-1

**Published:** 2025-12-30

**Authors:** Tao Yin, Metin Yesiltepe, Sanjay Metkar, Aubin Ramon, Matthew Greenig, Pietro Sormanni, Luciano D’Adamio

**Affiliations:** 1 Department of Pharmacology, Physiology & Neuroscience New Jersey Medical School, Brain Health Institute, Jacqueline Krieger Klein Center in Alzheimer’s Disease and Neurodegeneration Research, The State University of New Jersey, 205 South Orange Ave, RutgersNewark, NJ 07103 USA; 2 Centre for Misfolding Diseases, Department of Chemistry, Yusuf Hamied, University of Cambridge, Lensfield Road, Cambridge, CB2 1EW UK; 3 NanoNewron LLC, Townsend Hall T217, 1000 Morris Avenue, Union, NJ 07083 USA

**Keywords:** TNFα, Nanobody, Blood–brain-barrier, Alzheimer Disease, TNFα inhibition, Single-domain antibody

## Abstract

**Background:**

Delivery of biologic therapeutics to the central nervous system (CNS) is hindered by the blood–brain barrier (BBB), which restricts large molecule passage. Receptor-mediated transcytosis via transferrin receptor 1 (TfR1) provides a physiological route for selective BBB transport. This study aimed to develop human-specific nanobodies that engage TfR1 without disrupting transferrin function, enabling safe and efficient CNS delivery of therapeutic biologics.

**Methods:**

Single-domain camelid antibodies targeting human TfR1 were isolated, humanized, and optimized through computational and artificial intelligence–guided algorithms to improve humanness, solubility, and stability. Binding kinetics were quantified by surface plasmon resonance using a 1:1 Langmuir model. In vivo BBB permeability and safety were assessed in rats genetically humanized for TfR1 and transferrin following intravenous or subcutaneous administration.

**Results:**

Optimized TfR1-binding nanobodies exhibited high affinity for human TfR1, with equilibrium dissociation constants (KD) in the picomolar range. These nanobodies crossed the BBB efficiently without interfering with transferrin binding or iron homeostasis and were therefore designated NewroBus. When fused to humanized tumor necrosis factor alpha (TNFα)–neutralizing nanobodies, NewroBus heterodimers maintained BBB permeability and achieved sustained cerebrospinal fluid and serum levels for at least three days after subcutaneous dosing. Chronic administration of representative constructs in humanized rats did not alter hematologic parameters, indicating absence of TfR1-related hematotoxicity.

**Conclusions:**

Humanized TfR1 nanobodies (NewroBus) enable efficient, TfR1-dependent transcytosis of biologics across the BBB while preserving iron transport and safety. Their high binding affinity, favorable pharmacokinetic properties, and modular fusion capacity position NewroBus as a versatile platform for CNS delivery of therapeutic proteins.

**Supplementary Information:**

The online version contains supplementary material available at 10.1186/s12964-025-02605-1.

## Introduction

TfR1 is abundantly expressed on brain endothelial cells, where it mediates receptor-dependent transcytosis of iron-bound transferrin. This physiological transport mechanism has been increasingly exploited to deliver therapeutic molecules across the BBB, representing a rapidly advancing area in CNS drug delivery [1–3]. Several biotechnology platforms have emerged to harness TfR1-mediated transcytosis, including Denali Therapeutics’ Transport Vehicle (TV) based on engineered Fc domains that bind human TfR1 [4], Roche’s Brain Shuttle bispecific antibody approach [5], and Apertura Gene Therapy’s engineered AAV capsids designed for TfR1 targeting [6]. BioArctic has also announced a TfR1-targeting Brain Transporter™ platform, although detailed preclinical or clinical validation has not yet been disclosed. Among TfR1-targeted approaches, IZCARGO is the only clinically approved anti-TfR1 therapeutic [7].

Other emerging platforms, such as ALIA-1758 based on the MODEL™ technology, have also been reported; this approach is thought to leverage binders for both TfR1 and CD98 to transport diverse cargos across the BBB, although detailed mechanistic information remains limited.

One of the main potential drawbacks when targeting TfR1 is the potential interference with cellular iron uptake, because TfR1 is a major receptor for transferrin (TF). TF is the major ferric iron transport protein, which binds ferric (Fe3^+^) ions. The iron binding affinity of transferrin is pH dependent. In a neutral pH environment, TF (apotransferrin) binds iron with high affinity to form iron-bound TF (holotransferrin). In an acidic pH environment, the affinity of iron bound to transferrin decreases, dissociating iron from holotransferrin and releasing it into the environment. The importance of holotransferrin is to sequester Fe3^+^ ions in a relatively nonreactive and inert state to ensure normal free iron homeostasis in the body. Holotransferrin delivers iron to cells by binding to TfR1 (as well as TfR2). Neutral pH at the cell surface promotes binding of holotransferrin to TfR1/TfR2. The receptor-ligand complex enters the cell through receptor-mediated endocytosis and is internalized into endosomes. Relatively lower endosomal pH results in the release of iron. The receptor-ligand complex is recycled to the cell surface, where apotransferrin dissociates from TfR1/TfR2 [8]. Interference with these processes might lead to unintended toxic effects in patients treated with therapeutics based on TfR1b-Nbs. Thus, it is not surprising that interfering with TF-TfR1 interaction and/or uptake can have toxic effects, especially anemia. Homozygous null *Tfrc* mice display an embryonic lethal phenotype, while hypo-transferrinemic mice suffer severe anemia [9].

Camelids naturally produce heavy-chain-only antibodies that lack light chains and consist of a single variable domain (VHH) followed by constant domains CH2 and CH3 [10]. The isolated VHH domains, commonly referred to as nanobodies (Nbs), are compact, stable antigen-binding fragments with a typical molecular weight of 12–14 kDa. Compared to conventional monoclonal antibodies (mAbs), nanobodies offer several advantages: (1) their convex paratopes allow binding to recessed or cryptic epitopes, such as receptor binding pockets that are often inaccessible to the flat paratopes of mAbs; (2) they exhibit high stability across a wide pH range; (3) they have low immunogenicity, which can be further minimized through humanization or deimmunization; and (4) their small size and structural robustness make them more amenable to alternative delivery routes. Importantly, nanobodies targeting TfR1 have been shown to mediate transcytosis across the BBB [11, 12]. Additionally, nanobody-based TfR1 binders are monovalent and thus less likely to interfere with the physiological function of TfR1 in iron transport. Unlike bivalent antibodies, they are less likely to disrupt the transferrin (TF)–TfR1 interaction essential for cellular iron uptake or to promote TfR1 endocytosis, which typically requires engagement of both subunits of the TfR1 homodimer. This minimizes the risk of iron deficiency-related toxicities, an important consideration in the development of TfR1-targeted therapeutics.

Based on these properties, we set out to identify and characterize novel anti-TfR1 nanobodies capable of efficiently crossing the BBB, with the long-term goal of using them as “Trojan horses” to deliver otherwise BBB-impermeable therapeutics into the brain.

## Methods

### Animals

All animal procedures were conducted in accordance with the NIH *Ethical Guidelines for the Treatment of Laboratory Animals*. All protocols were approved by the Rutgers Institutional Animal Care and Use Committee (IACUC Protocol #201,702,513). Efforts were made to minimize animal suffering and reduce the number of animals used.

Cell lines

**Table:** 

Cell Line	Description	Vendor	Catalog No
HEK293T	Human embryonic kidney cells with SV40 large T-antigen	ATCC	CRL-3216
WEHI-13VAR	Mouse fibrosarcoma TNF-sensitive reporter cell line	ATCC	CRL-2168
HEK-ATP089	HEK293 cells stably expressing human TfR1	Acro Biosystems	CHEK-ATP089

Expression constructs

**Table:** 

Construct	Description	Vendor/Source	Catalog No. (VB)
Human TfR1 + EGFP	Mammalian expression vector for human TfR1 + EGFP	VectorBuilder	VB211221-1144bue
Mouse Tfr1 + EGFP	Mammalian expression vector for mouse Tfr1 + EGFP	VectorBuilder	VB220126-1168dfv
Rat Tfr1 + EGFP	Mammalian expression vector for rat Tfr1 + EGFP	VectorBuilder	VB220126-1169wgz

These constructs can be retrieved from VectorBuilder’s plasmid lookup portal: https://en.vectorbuilder.com/design/retrieve.html

Primary antibodies

**Table:** 

Target	Antibody Description	Vendor	Catalog No
His tag	Anti-His tag (APC-conjugated)	R&D Systems	IC050A
His tag	Anti-His tag (Alexa Fluor 488-conjugated)	R&D Systems	IC050G
His tag	Rabbit mAb	Cell Signaling Technology (CST)	12,698
His tag	Alexa Fluor 488 Rabbit mAb (D3I1O)	CST	14,930
VHH domain	Goat Anti-Alpaca IgG, VHH domain	Jackson ImmunoResearch	128–005–230
VHH domain (biotinylated)	Biotinylated VHH domain	Jackson ImmunoResearch	128–065–232
Human TfR1	APC-conjugated monoclonal	Invitrogen	17,071,942
Mouse Tfr1	APC-conjugated monoclonal	Invitrogen	17,071,182
Rat Tfr1	APC-conjugated monoclonal	Invitrogen	17,071,082
Human TfR1	Mouse antibody	CST	54505S
Iba1 (microglia)	SPICA Dye™ 594-conjugated Rabbit antibody	Wako (FUJIFILM)	012–28401
GFAP (astrocytes)	Mouse monoclonal	BD Biosciences	556,327
Glut1	Rabbit monoclonal	CST	73,015
GAPDH	Rabbit monoclonal	Sigma-Aldrich	G9545
MBP (oligodendrocytes)	Rabbit monoclonal	CST	78,896
NMDA Receptor 2	Rabbit monoclonal	CST	4212
EAAT2 (astrocytes)	Rabbit polyclonal	Synaptic Systems	250,203, 104,202

Secondary antibodies

**Table:** 

Target	Antibody Description	Vendor	Catalog No
Goat IgG	Anti-Goat IgG Donkey SULFO-TAG	Meso Scale Discovery (MSD)	R32AG
Rabbit IgG	Anti-Rabbit IgG Donkey SULFO-TAG	MSD	R32AB
Mouse IgG	Goat anti-Mouse IgG (H + L), Alexa Fluor™ 594, HCA	Invitrogen	A-11032
Rabbit IgG	HRP-conjugated secondary antibody	CST	7074
Rabbit IgG	HRP-conjugated secondary antibody	Southern Biotech	OB405005

Recombinant proteins and detection reagents

**Table:** 

Protein/Reagent	Description	Vendor	Catalog No
Human TfR1	Biotinylated recombinant protein	Acro Biosystems	TFR-H82E5
Human TNFα	Recombinant active trimer	Acro Biosystems	TNA-H5228
Human Transferrin	Unlabeled	Jackson ImmunoResearch	009000050
Human Transferrin (FITC-conjugated)	Fluorescently labeled	Jackson ImmunoResearch	009090050
Human Transferrin (pHrodo™ Red-labeled)	pHrodo-conjugated transferrin	Thermo Fisher Scientific	P35376
Biotinylated TNFα	ELISA capture antigen	Acro Biosystems	TNA-H82E3-25ug
Biotinylated His Tag	ELISA control	R&D Systems	BAM050
Biotinylated VHH Domain	ELISA control	Jackson ImmunoResearch	128–065–232

General reagents and lab chemicals

**Table:** 

Reagent	Description	Vendor	Catalog No
Fugene® HD Transfection Reagent	Non-liposomal transfection reagent	Promega	E2311
Fetal Bovine Serum (FBS)	Heat-inactivated, cell culture supplement	Gibco	A3840102
EDTA	Chelating agent (disodium salt)	Sigma-Aldrich	N6507
DMEM Medium	High-glucose cell culture medium	Corning	10–017-CV
RPMI 1640 Medium	Cell culture medium for lymphoid cells	Corning	10–040-CV
Acridine Orange/Propidium Iodide (AO/PI)	Dual dye viability stain	DeNovix	CD-AO-PI-7.5
Propidium Iodide (PI)	DNA-binding dye for dead cell discrimination	Invitrogen	P3566
Actinomycin D	RNA synthesis inhibitor for cytotoxicity assays	Sigma-Aldrich	A9415
Caspase-3/7 Green Detection Reagent	Fluorescent apoptosis marker	Thermo Fisher Scientific	C10423
Dextran (MW 60,000)	Used in brain fractionation	Sigma-Aldrich	31,397
Hygromycin B	selection marker	Sigma-Aldrich	H3272
S1 Buffer Components	Tris base, sucrose, EDTA, EGTA (lab-prepared)		

Consumables and tools for sample collection

**Table:** 

Item	Description	Vendor
1 mL Tuberculin Syringe, 25G	For IV injection	Cardinal Health
VACUETTE Blood Collection Set, 25G	For tail vein blood collection	Greiner Bio-One
BD Microtainer SST Tubes	For tail vein serum isolation	Becton Dickinson
BD Vacutainer SST Tubes	For terminal serum isolation	Becton Dickinson
Air-Tite Syringe, 5 mL	For cardiac blood collection	Air-Tite Products Co., Inc
Monoject Hypodermic Needle, 21G	For cardiac blood collection	Cardinal Health
EXEL Insulin Syringe, 28G	For CSF collection	EXEL
Stereotaxic Frame	For CSF collection stabilization	Stoelting Co
MiniCollect K2EDTA Tubes	For CBC analysis	Greiner Bio-One

Histology and microscopy reagents

**Table:** 

Reagent	Description	Vendor	Catalog No
Paraformaldehyde, 4%	Tissue fixative	Electron Microscopy Sciences	15,714-S
30% Sucrose Solution	Cryoprotection	Lab-prepared	—
OCT Embedding Compound	Tissue embedding medium	Fisher	23–730-571
Superfrost Plus Glass Slides	Charged slides for cryosections	Fisher	22–037–246
Coverslips, 22 × 50 mm	For mounting stained sections	Corning	2980–225
Triton X-100	Detergent for permeabilization	Sigma-Aldrich	T9284
Super PAP Pen	Hydrophobic barrier pen for IHC	IHCWORLD	SPM0928
DAPI Mounting Medium	Antifade mounting with nuclear stain	Southern Biotech	0100–20

Instrumentation and software

**Table:** 

Instrument/Software	Description	Vendor
MESO QuickPlex SQ 120	Electrochemiluminescence plate reader	Meso Scale Discovery
Incucyte Live-Cell Analysis System	Real-time imaging for cell-based assays	Sartorius
Trans-Blot Turbo System	Protein blotting system	Bio-Rad
ChemiDoc MP Imaging System	Gel and blot imaging	Bio-Rad
Image Lab Software	Western blot quantification	Bio-Rad
GraphPad Prism	Data analysis and statistics	GraphPad
Biacore 1 K SPR System	Surface plasmon resonance (SPR) analysis	Cytiva
Nikon A1R + HD Confocal Microscope	Fluorescence imaging with Z-stack acquisition	Nikon
Leica CM1950 Cryostat	Rotary cryostat for the sectioning of frozen tissue specimen	Leica
NIS Element Viewer	Confocal image processing	Nikon

### Generation and screening of camelid anti-TfR1 nanobodies

To generate anti-TfR1 nanobodies (anti-TfR1-Nbs), one alpaca and one llama were immunized with the human TfR1 ectodomain recombinant protein (Acro Biosystems, TFR-H82E5). The immunization protocol began with an initial subcutaneous injection of 0.5 mg TfR1 mixed with Complete Freund’s Adjuvant (CFA) at week 0, followed by booster injections of 0.5 mg TfR1 with Incomplete Freund’s Adjuvant (IFA) every two weeks up to week 14. Serum samples were collected before immunization and at designated time points to assess antibody titers via ELISA, utilizing TfR1-coated plates with appropriate negative and positive controls to ensure specificity.

At weeks 10 and 14, 500 mL of whole blood were drawn from each llama for peripheral blood mononuclear cell (PBMC) isolation. PBMCs were isolated within four hours of blood collection using density gradient centrifugation, ensuring cell viability above 99% as determined by trypan blue exclusion. Total RNA was extracted from the isolated PBMCs using the RNeasy Maxi Kit (Qiagen) and quantified by spectrophotometry, ensuring an A300/A280 ratio greater than 1.9. High-quality RNA was confirmed by agarose gel electrophoresis, displaying distinct 18S and 28S rRNA bands without signs of degradation. Complementary DNA (cDNA) was synthesized from the purified RNA using the SuperScript IV First-Strand Synthesis System (Thermo Fisher Scientific). A nanobody-specific library was constructed by amplifying the variable regions of heavy-chain-only antibodies (VHH) through a two-step PCR process using camelid-specific degenerate primers. The amplified VHH fragments were cloned into the pADL-20c phagemid vector using SfiI restriction sites and transformed into E. coli TG1 cells, resulting in a phage display library containing approximately 2.57 × 10⁹ individual clones. Library diversity was confirmed by sequencing 74 random clones, which revealed 88% contained VHH inserts with intact open reading frames and no duplicate sequences. Library panning was performed against immobilized TfR1 through three rounds of selection to enrich for specific binders. To reduce non-specific interactions, the phage pool was pre-absorbed on BSA-coated wells before each panning round. Enrichment of specific phage binders was monitored using dot assays, which demonstrated increased binding to TfR1 with each successive round.

Following panning, 470 individual clones were screened using an off-phage ELISA to identify those with specific binding to TfR1 and minimal binding to BSA. Positive clones were further validated through repeat screening and sequencing to ensure specificity and diversity. Selected nanobodies were expressed in a non-amber-suppressor strain of E. coli, and periplasmic fractions containing His-tagged nanobodies were purified using His-tag affinity chromatography. Purity was confirmed by SDS-PAGE, and nanobody concentrations were determined by absorbance at 280 nm. Purified nanobodies were dialyzed into PBS (pH 7.4) and filter-sterilized for downstream applications.

### FACS analysis using transfected HEK293T cells and stable HEK293-hTfR1 cells

HEK293T cells (ATCC, CRL-3216) were cultured in DMEM (Corning, 10–017-CV) supplemented with 10% fetal bovine serum (Gibco, A3840102) at 37 °C in a humidified 5% CO₂ incubator. For transient expression of transferrin receptor 1 (TfR1), cells were transfected with plasmids encoding human TfR1-EGFP, mouse Tfr1-EGFP, or rat Tfr1-EGFP (VectorBuilder; VB211221-1144bue, VB220126-1168dfv, and VB220126-1169wgz, respectively) using Fugene HD transfection reagent (Promega, E2311), according to the manufacturer’s protocol. Cells were harvested for analysis 24–48 h post-transfection. HEK293-hTfR1 cells (Acro Biosystems, CHEK-ATP089), a stable clone expressing human TfR1, were maintained under the same conditions and used as a positive control for TfR1 surface expression and nanobody binding. 100 μg/mL of Hygromycin B (Sigma, H3272) was used for specific selection of TfR1 expressed clones.

#### Staining procedure

Cells were resuspended in ice-cold FACS buffer (PBS containing 1% BSA and 1 mM EDTA, pH 7.2) and incubated with nanobodies (final concentration 40–100 nM) at 4 °C for 45 minutes with gentle shaking. After three washes in FACS buffer, cells were incubated for 30 minutes at 4 °C with APC-conjugated anti-His tag antibody (R&D Systems, IC050A) at 1:100 dilution to detect bound His-tagged nanobodies. Following additional washes, cells were stained with propidium iodide (Invitrogen, P3566; 1:1000 dilution) to exclude dead cells.

#### Flow cytometry analysis

Samples were analyzed on a BD LSR Fortessa or similar flow cytometer. Data were gated on live, EGFP-positive cells (for transfection marker) and analyzed for APC signal using FlowJo software. For comparative binding across species-specific TfR1, anti-human, anti-mouse, and anti-rat APC-conjugated antibodies (Invitrogen; Cat. Nos. 17071942, 17071182, 17071082) were also used in parallel at 1:100 dilution.

### TNFα inhibition and IC₅₀ determination using

WEHI-13VAR cells (ATCC, CRL-2168), a TNFα-sensitive mouse fibrosarcoma line, were cultured in RPMI 1640 medium (Corning, 10–040-CV) supplemented with 10% fetal bovine serum (Gibco, A3840102) at 37 °C with 5% CO₂. Cells were seeded into 96-well plates at 30,000 cells per well in RPMI containing 10% FBS and 1 μg/mL Actinomycin D (Sigma-Aldrich, A9415) to sensitize cells to TNFα-induced apoptosis. Recombinant human TNFα (Acro Biosystems, TNA-H5228) was pre-incubated with varying concentrations of TNFI nanobodies for 30 min at room temperature, then added to the cells at a final TNFα concentration of 0.25 ng/mL. Caspase-3/7 activity was monitored using Caspase-3/7 Green Detection Reagent (Thermo Fisher Scientific, C10423) added at a final concentration of 0.5 μM. Plates were immediately placed in the Incucyte Live-Cell Imaging System (Sartorius), and fluorescent signals were monitored every 2–4 h for up to 24 h. Fluorescent caspase-3/7 signal intensity was quantified using Incucyte analysis software. TNFα-induced apoptosis was set to 100%, and inhibition was expressed as a percentage of this maximum signal. IC₅₀ values for each TNFI-Nb were calculated using GraphPad Prism with nonlinear regression (log[inhibitor] vs. response – variable slope).

### Transferrin–TfR1 binding competition assay

HEK293-hTfR1 stable cells were cultured in DMEM (Corning, 10–017-CV) supplemented with 10% fetal bovine serum (Gibco, A3840102) and 100 μg/mL of Hygromycin B under standard conditions (37 °C, 5% CO₂). Cells were harvested at ~ 80% confluence, washed with ice-cold PBS, and resuspended in FACS buffer (PBS + 1% BSA + 1 mM EDTA).

#### Competition assay

Cells were incubated for 60 minutes at 4 °C in 96-well plates with the following conditions (in 200 µL final volume per well):

- TF-FITC alone: 2.5 µM FITC-conjugated human transferrin (Jackson ImmunoResearch, 009090050)
- Nanobody alone: 2.5 µM of individual TfR1b-Nbs
- TF-FITC + Nanobody: 2.5 µM of both reagents co-incubated
- TF-FITC + Unlabeled TF: Co-incubation with increasing concentrations of unlabeled human TF (30 nM, 750 nM, 2.5 µM, and 7.5 µM; Jackson ImmunoResearch, 009000050)

Following incubation, cells were washed 3× with FACS buffer and resuspended in buffer containing 1:1000 dilution of propidium iodide (Invitrogen, P3566) to exclude dead cells.

#### Flow cytometry analysis

Fluorescence was measured using a flow cytometer. FITC signal was gated on live cells, and the mean fluorescence intensity (MFI) of the FITC channel was used to quantify TF-FITC binding. Competitive inhibition was determined by comparing MFI between conditions. Decreased MFI in the presence of excess unlabeled TF or TfR1b-Nbs indicated interference with TF-TfR1 binding.

### Transferrin uptake interference assay using incucyte live-cell imaging

HEK293-hTfR1 stable cells were plated in black-walled 96-well tissue culture plates at a density of 30,000 cells per well in DMEM (Corning, 10–017-CV) with 10% fetal bovine serum (Gibco, A3840102) and 100 μg/mL of Hygromycin B. After 24 h, cells reached approximately 80% confluence and were ready for assay. To assess TfR1-mediated uptake, pHrodo™ Red-conjugated human transferrin (Thermo Fisher Scientific, P35376) was used at a final concentration of 312.5 nM. Uptake competition was evaluated by co-incubating with either:

Unlabeled human transferrin at increasing concentrations (30 nM, 150 nM, 750 nM, and 3750 nM), or, selected TfR1b-Nbs at 40 nM.

All reagents were diluted in serum-free DMEM. Cells were incubated at 37 °C immediately following reagent addition.

#### Live imaging and quantification

Plates were placed into the Incucyte® Live-Cell Analysis System (Sartorius), and images were acquired every 1–2 hours over a 24-hour period using the red fluorescence channel (excitation/emission ~560/585 nm). The pHrodo dye emits fluorescence only in acidic intracellular compartments, allowing real-time tracking of transferrin uptake via receptor-mediated endocytosis.

#### Data analysis

Total integrated fluorescence intensity per well was quantified using Incucyte software. Uptake inhibition by TfR1b-Nbs or unlabeled transferrin was calculated as the percent decrease in red signal compared to wells treated with pHrodo-TF alone. All conditions were performed in triplicate and repeated in independent experiments.

### In vivo drug administration, perfusion, and sample collection

#### Intravenous (IV) injection

Rats were briefly warmed under a heat lamp to promote vasodilation. Intravenous drug administration was performed via the lateral tail vein using 1 mL Tuberculin Syringes fitted with 25G needles (Cardinal Health). Nanobodies or fusion constructs were injected slowly at a volume of 1 µL per gram of body weight in sterile PBS to minimize stress and ensure accurate dosing.

#### Subcutaneous (SQ) injection

For SQ administration, animals were gently restrained using a towel, and the compound was injected into the interscapular area using a 1mL Tuberculin syringes fitted with 25G needles (Cardinal Health). Dosing volume was the same as IV: 1 µL of a 40 µM solution per gram of body weight in PBS. Animals were monitored for signs of discomfort or inflammation at the injection site.

#### Perfusion and brain tissue collection

At the designated time points post-injection, animals were deeply anesthetized using isoflurane. For terminal experiments involving brain collection, transcardiac perfusion was performed with cold PBS (without calcium or magnesium) at a flow rate of 10 mL/min for 10 minutes to remove blood from the vasculature. Brains were immediately extracted and processed for either biochemical assays or immunohistochemistry.

#### Serum collection

##### Non-terminal

 Blood samples (~300 µL) were collected via tail vein using a 25G VACUETTE Safety Blood Collection Set (Greiner Bio-One) into BD Microtainer Serum Separator Tubes (Becton Dickinson). Samples were allowed to clot for 30 minutes at room temperature, followed by centrifugation at 9000 × g for 30 seconds.

##### Terminal

Blood was collected by cardiac puncture using a 5 mL syringe (Air-Tite Products Co., Inc) fitted with a 21G needle (Cardinal Health), transferred into BD Vacutainer SST tubes, allowed to clot for 30 minutes at room temperature, and centrifuged at 2000 × g for 10 minutes. All serum samples were aliquoted and stored at –80°C.

#### CSF collection

Following transcardiac perfusion and prior to brain tissue collection, CSF was collected via cisterna magna puncture. Animals were placed in a stereotaxic frame (Stoelting Co.). The head was flexed forward, and a midline incision was made at the base of the skull to expose the translucent dura mater over the cisterna magna, using a 28G insulin syringe (EXEL), CSF was slowly withdrawn, carefully avoiding blood contamination. Typically, 50–80 µL of CSF was collected per animal. Samples were snap-frozen in liquid nitrogen and stored at –80°C.

### ELISA for TfR1- and TNFα-based detection of nanobodies

#### TfR1-based ELISA

Streptavidin-coated 96-well plates (Meso Scale Discovery, L45SA) were blocked overnight at 4 °C with 3% BSA in PBST (PBS + 0.05% Tween-20). Plates were then coated with 0.25 µg/mL biotinylated human transferrin receptor 1 (TfR1) protein (Acro Biosystems, TFR-H82E5) in PBS for 1 h at room temperature with shaking.

After washing 4× with PBST, samples containing nanobodies were added and incubated overnight at 4°C. Following three PBST washes, wells were incubated with 1 µg/mL Goat Anti-Alpaca IgG, VHH domain (Jackson ImmunoResearch, 128-005−230) for 1 hour at room temperature. After additional washes, SULFO-TAG-labeled anti-goat IgG secondary antibody (Meso Scale Discovery, R32AG; 0.5–1 µg/mL) was added and incubated for 1 hour.

#### TNFα-based ELISA

The same procedure was used with the following modifications: plates were coated with 0.2 µg/mL biotinylated human TNFα (Acro Biosystems, TNA-H82E3-25ug) instead of TfR1. Detection of nanobody binding was performed using either anti-His tag rabbit monoclonal antibody (Cell Signaling Technology, 12698; 1:500 dilution) or Goat Anti-Alpaca IgG VHH domain antibody, depending on the tag configuration. Corresponding SULFO-TAG-labeled anti-rabbit (R32AB) or anti-goat (R32AG) secondary antibodies were used.

#### Plate reading and analysis

After final washes, wells were developed using 2× MSD Read Buffer (Meso Scale Discovery, R92TC) and read on a MESO QuickPlex SQ 120 instrument. Background-subtracted signals were normalized to control wells. TNFI-Nb1 (specific for TNFα) and irrelevant control nanobodies were used to confirm specificity.

### Hematotoxicity assessment via Complete Blood Count (CBC)

To evaluate hematotoxicity following intravenous administration of TfR1-targeting nanobody constructs, complete blood counts (CBCs) were performed in treated and control rats at multiple time points. Animals received IV injections of either PBS (Group 1, *n* = 7; 3 males, 4 females) or TNFI-β–TfR1b–A2 (Group 2, *n* = 8; 3 males, 5 females) at a dose of 1 µL of a 40 µM solution per gram of body weight. Prior the blood collection, rats were briefly warmed under a heat lamp. Blood samples were collected from the tail lateral vein using a VACUTTE Safety Blood Collection Set (Greiner Bio-One) and transferred into MiniCollect K2EDTA tubes (Greiner Bio-One). Samples were immediately placed on an icepack and kept at + 4 °C until analysis and transported to the In Vivo Research Services (IRVS) Core Facility at Rutgers University. CBCs were performed using the Heska Element HT5 CBC Analyzer.

CBCs were collected at four time points:

- Day –3 (D–3): Baseline, prior to treatment
- Day 1 (D1): 24 h after the first injection
- Day 17 (D17): After the third injection
- Day 24 (D24): After the fourth injection

The following parameters were measured:

White blood cell (WBC) profile:

- Total WBC (×10³/μL)
- Neutrophils (NEU), Lymphocytes (LYM), Monocytes (MONO), Eosinophils (EOS), Basophils (BAS)
- Percent distribution: NEU%, LYM%, MONO%, EOS%, BAS%

Red blood cell (RBC) profile:

- RBC count (× 10⁶/μL), Hemoglobin (HGB, g/dL), Hematocrit (HCT, %)
- Mean corpuscular volume (MCV, fL), Mean corpuscular hemoglobin (MCH, pg)
- Mean corpuscular hemoglobin concentration (MCHC, g/dL)
- Red cell distribution width (RDW, %)

These data were used to monitor for treatment-related hematologic changes, particularly indicators of anemia, leukocyte shifts, and thrombocytopenia.

### Fractionation of brain tissue

To evaluate the distribution of nanobodies between the brain vasculature and parenchyma, rats were deeply anesthetized and perfused transcardially with cold PBS. The right hemisphere was dissected, and choroid plexuses were removed. Tissue was homogenized in 5 mL of ice-cold S1 buffer (250 mM sucrose, 20 mM Tris-base pH 7.4, 1 mM EDTA, 1 mM EGTA) using a glass-Teflon homogenizer (10 strokes). Homogenates were centrifuged at 1,000 × g for 10 minutes at 4°C. Pellets were resuspended in 2 mL of 17% dextran (MW 60,000; Sigma, 31397) and centrifuged at 4,200 × g for 15 minutes. The pellet was collected as the capillary-enriched vasculature fraction. The supernatant was diluted in S1 buffer and centrifuged again at 4,200 × g for 15 minutes. The resulting pellet was collected as the vascular-depleted parenchymal fraction.

Both fractions were lysed in ice-cold S1 buffer containing protease and phosphatase inhibitors and sonicated (50% amplitude, 3 × 10 s bursts with 30 s interval rests). Protein concentrations were determined by Bradford assay. Aliquots were used for ELISA and Western blot.

## Western blotting of brain fractions

Equal amounts of protein from homogenate, vasculature, and parenchymal fractions were mixed with LDS sample buffer (Invitrogen, NP0007) containing 10% β-mercaptoethanol, heated at 95 °C for 5 min, and loaded onto 4–12% Bis–Tris polyacrylamide gels (Bio-Rad, 3,450,125). Electrophoresis was followed by transfer to nitrocellulose membranes using the Trans-Blot Turbo System (Bio-Rad) at 25 V for 7 min. Membranes were blocked with 5% non-fat milk (Bio-Rad, 1,706,404) in PBST (PBS + 0.05% Tween-20) for 45 min, then incubated overnight at 4 °C with primary antibodies (1:1000 dilution in blocking buffer):

- Anti-human TfR1 (CST, 13,113)
- Anti-Glut1 (CST, 73,015) – endothelial cell marker
- Anti-GAPDH (Sigma, G9545) – loading control
- Anti-VAMP2, NMDAR2B (CST, 4212) – neuronal markers
- Anti-IBA1 (Wako, 01620001) – microglial marker
- Anti-EAAT2 (Synaptic Systems, 250,203 or 104,202) – astrocytic marker
- Anti-MBP (CST, 78,896) – oligodendrocyte marker

After washing, membranes were incubated for 45 minutes at room temperature with HRP-conjugated secondary antibodies (anti-rabbit: CST 7074 or Southern Biotech OB405005, 1:1000 in 5% milk). Detection was performed using Clarity ECL substrate (Bio-Rad, 1705061), and bands were visualized with the ChemiDoc MP Imaging System (Bio-Rad). Densitometry analysis was performed using Image Lab software.

These blots validated the separation of vascular and parenchymal compartments and confirmed human TfR1 expression and nanobody localization across fractions.

### Immunohistochemistry (IHC)

Rats were deeply anesthetized and perfused transcardially with PBS (without calcium and magnesium), followed by fixation with 4% paraformaldehyde (PFA; Electron Microscopy Sciences, 15,714-S) in PBS at a flow rate of 10 mL/min for 10 min. Brains were extracted and post-fixed in 4% PFA at 4 °C for 24 h on a shaker. After fixation, brains were washed twice in PBS and incubated in 30% sucrose in PBS at 4 °C for 48 h for cryoprotection. Brains were embedded in OCT compound (Fisher, 23–730-571), frozen, and stored at –80 °C. Coronal Sects. (20 μm thick) were cut on a cryostat (Leica CM1950) and mounted onto charged glass slides (Fisher, 22–037–246), then stored at –80 °C until use.

#### Immunostaining procedure

Slides were brought to room temperature, and a hydrophobic barrier was drawn around each section using a Super PAP Pen. Sections were rehydrated in PBS for 10 minutes and blocked in 10% normal goat serum containing 0.3% Triton X-100 (Sigma, T9284) for 1 hour at room temperature.

Sections were incubated overnight at 4 °C with the following primary antibodies diluted in 5% serum with 0.3% Triton X-100:

**Table:** 

Primary Antibody	Target	Dilution	Vendor/Cat. No
His-tag (Alexa Fluor 488)	Nanobody localization	1:250	CST, 14,930
Anti-IBA1 (SPICA Dye 594)	Microglia	1:250	Wako, 012–28401
Anti-GFAP	Astrocytes	1:250	BD Biosciences, 556,327
Anti-human TfR1	Human TfR1	1:250	CST, 54505S

After three washes in PBS containing 0.3% Triton X-100, sections were incubated for 2 hours at room temperature with appropriate fluorescent secondary antibodies diluted 1:1000 in PBS with 5% serum and 0.3% Triton X-100. The following secondary antibody was used:

**Table:** 

Secondary Antibody	Target	Dilution	Vendor/Cat. No
Goat anti-Mouse IgG (Alexa Fluor 594)	GFAP, TfR1	1:1000	Invitrogen, A-11032

Sections were washed in PBS and mounted using DAPI-containing aqueous mounting medium (Southern Biotech, 0100-20) and cover slipped (Corning, 2980-225).

#### Imaging and analysis

Confocal imaging was performed using a Nikon A1R inverted laser-scanning confocal microscope. Z-stacks and tile scans were acquired using consistent settings across experimental conditions. Colocalization of nanobody signal with cell-type markers was assessed in the cortex and hippocampus. Image processing was performed using NIS element view.

#### SPR analyses

SPR analyses were performed by Rapid Novor to ensure standardized, high-quality kinetic measurements. The purity of recombinant human TfR1 and nanobodies TfR1b-A2 and TfR1b-D1 exceeded 90%, ensuring reliable data.

Kinetic studies were conducted on a Cytiva Biacore 1 K instrument equipped with carboxyl CM5 sensor chips. Ligands were immobilized in 10 mM acetate buffer (pH 5.5) at a flow rate of 20 µL/min for 210 seconds. Immobilization levels were 45.9 RU (Rmax 619.3 RU) for A2 and 20.6 RU (Rmax 271.5 RU) for D1, using ligand concentrations of 2.5 µg/mL and 1 µg/mL, respectively. The optimized running buffer consisted of PBS supplemented with 3 mM EDTA and 0.05% (v/v) Surfactant P20, which provided optimal stability and reproducibility for kinetic analysis.

Analytes were diluted in the same running buffer to generate five to eight concentrations in a 3-fold serial dilution series and injected from lowest to highest concentration at 30–40 µL/min. Association and dissociation phases were set at 120 seconds and 600 seconds, respectively. Sensor surfaces were regenerated after each cycle with a single injection of 10 mM glycine-HCl (pH 1.5).

Kinetic data were analyzed with Biacore Insights software using a 1:1 Langmuir binding model with global fitting. Sensorgrams showed specific, dose-dependent binding with residuals <10% of response and χ^2^ <10% Rmax, confirming high fit quality. The equilibrium dissociation constants (KD) were in the high-picomolar range, with A2 showing slightly higher affinity to human TfR1 than D1.

### Statistical analysis

All quantitative data are presented as mean ± standard error of the mean (SEM), unless otherwise indicated. Statistical analyses were performed using GraphPad Prism software. Comparisons between two groups were made using unpaired two-tailed Student’s t-tests. Mann–Whitney test, a non-parametric method that does not assume normal distribution and is well suited for comparing two independent groups when the data may be skewed or non-normally distributed, has been used for data shown in Fig. 2a. For multiple group comparisons, one-way or two-way ANOVA followed by appropriate post hoc tests (e.g., Tukey’s or Sidak’s) were used. A *p*-value < 0.05 was considered statistically significant. The number of biological replicates (n) is indicated in the figure legends or methods.

## Results

### Identification of TfR1b-Nbs

Four hundred seventy individual VHH domain clones isolated from PBMCs from 1 llama and 1 alpaca immunized with human TfR1 extracellular domain (Sino Biological, HPLC-11020-H07H) were tested for antigen binding. 106 unique α-TfR1-Nb VHH sequences were identified. The above experiments were performed at Abcore.

**Fig. 1:**
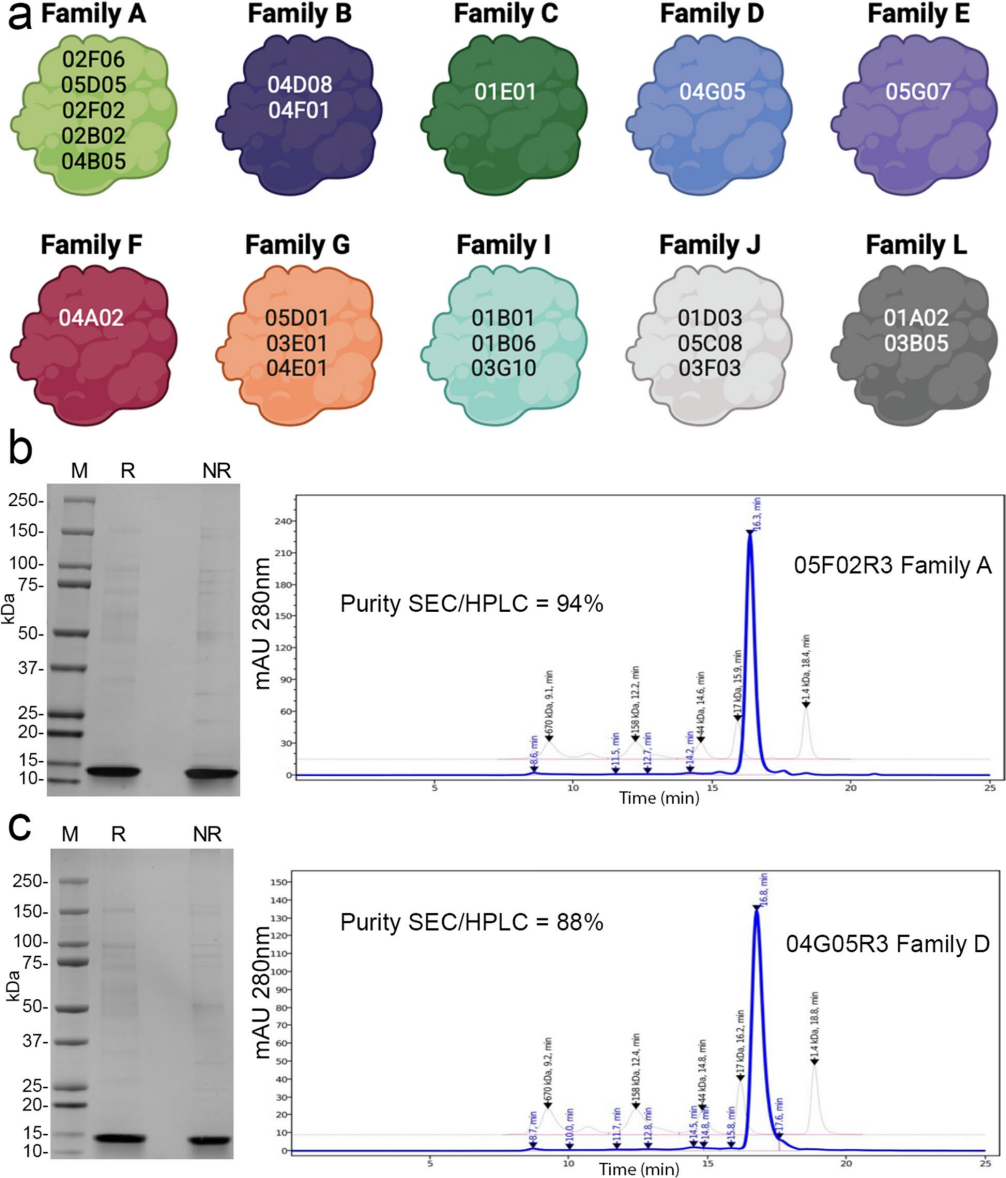
Protein purification and analysis of selected TfR1b-Nbs. **a** Schematic representation of the 24 TfR1b-Nbs produced in mammalian cells. **b** SDS-PAGE and SEC-HPLC analysis of TfR1b-Nb 05F02R3 (Family A). Coomassie-stained SDS-PAGE shows M = molecular weight marker; Lane 1 = purified 02B02R3 under reducing conditions; Lane 2 = under non-reducing conditions. Left panel: SEC-HPLC profile of purified 05F02R3. **c** SDS-PAGE and SEC-HPLC analysis of TfR1b-Nb 04G05R3 (Family D), shown as in (B)

HEK293 human cells were transfected with a vector co-expressing human TfR1 and EGFP. Of the 106 α-TfR1 nanobodies (Nbs) produced in bacteria, 24 bind to cell-surface hTfR1 on transfected cells (Supporting Figures S1, S2, S3 and S4). These nanobodies were designated TfR1b-Nbs (TfR1-binding nanobodies). None of the 24 TfR1b-Nbs cross-reacted with rodent (rat or mouse) Tfr1 (Supporting Figures S5, S6, S7 and S8). Based on the complementarity-determining regions (CDRs) identities, these TfR1b-Nbs were grouped into ten families (Fig. 1a).

### Assessing the binding of TfR1b-Nbs produced in mammalian cells to human TfR1

Chinese Hamster Ovary (CHO) cells are a preferred system to produce therapeutic proteins due to their ability to generate complex, properly folded proteins with significantly lower endotoxin levels compared to bacterial expression systems, enhancing both the safety and clinical suitability of the final product. CHO-derived biologics have a well-established track record of regulatory approval, making this platform highly reliable for biopharmaceutical manufacturing.

The data in Table 1 and Fig. 1b and c (showing representative results for 05F02R3 from Family A and 04G05R3 from Family D) demonstrate the high purity and yield of TfR1b-Nbs produced with transient transfection in CHO-S cells, supporting their suitability as a production platform for preclinical and clinical development. Protein production was performed at GenScript.

**Table 1:** Production of TfR1b-Nbs in mammalian cells. It was performed as for TNFI-Nbs. The table shows quantity and purity of TfR1b-Nb purified from 100 ml CHO-S cell culture supernatants

Name	Family	Conc (mg/ml)	Purity SDS-PAGE (%)	Purity SEC-HPLC (%)	Endotoxin Level (EU/mg)	Total (mg)
02F06R3	A	0.45	75	92	0.111	~ 6.525
05D05R3	A	0.44	85	94	0.114	~ 6.38
05F02R3	A	0.46	90	94	0.109	~ 6.67
02B02R3	A	0.52	90	95	0.096	~ 7.54
04B05R3	A	0.53	50	96	0.094	~ 7.685
04D08R3	B	0.46	95	91	0.109	~ 6.67
04F01R3	B	0.42	80	89	0.119	~ 6.09
04G05R3	D	0.42	90	88	0.119	~ 6.09
05G07R3	E	0.29	70	83	0.172	~ 2.75
04A02R3	F	0.67	70	88	0.075	~ 9.045
05D01R3	G	0.6	75	89	0.083	~ 8.4
03E01R3	G	0.81	80	90	0.062	~ 11.34
04E01R3	G	0.88	70	92	0.057	~ 12.58
05D04R3	J	0.36	85	95	0.139	~ 5.04
01D03R3	J	0.84	75	94	0.064	~ 12.18
05C08R3	J	0.68	70	91	0.074	~ 9.18
01B03R3	J	0.73	80	94	0.068	~ 10.22
03F03R3	J	0.79	90	93	0.063	~ 11.06
01B01R3	I	0.92	95	96	0.054	~ 12.88
01B06R3	I	0.58	75	86	0.086	~ 8.7
03G10R3	I	0.79	85	88	0.063	~ 11.85
03B05R3	L	0.85	85	84	0.059	~ 12.75
01A02R3	L	0.72	70	83	0.069	~ 10.8

**Fig. 2:**
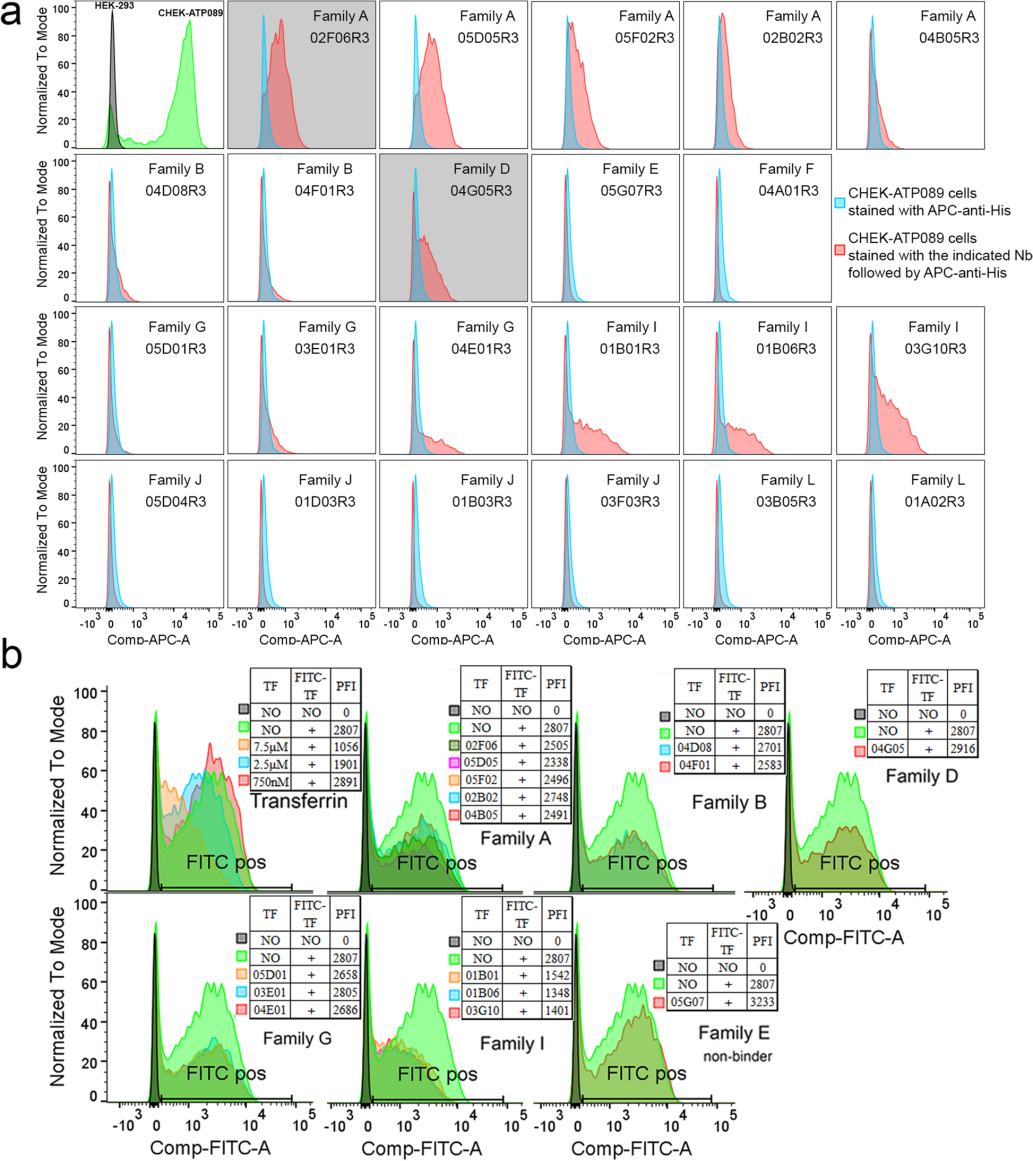
Selection of TfR1b-Nbs produced in mammalian cells that do not interfere with TF–TfR1 interaction. **a** The upper-left panel shows the specific binding of human Transferrin-FITC (at a concentration of 2.5 nM) to CHEK-ATP089 cells, with no binding observed on HEK293 cells. The remaining panels depict the binding of each TfR1b-Nb at a concentration of 40 nM to CHEK-ATP089 cells. No binding was observed on HEK293 cells. The two nanobodies highlighted in grey are the parental sequences from which the final selected NewroBus molecules were derived. **b** The upper-left panel demonstrates the dose-dependent competition of unlabeled Transferrin for binding to TfR1 on the cell surface of CHEK-ATP089 cells in the presence of FITC-Transferrin. Subsequent panels assess the inhibitory activity of 14 TfR1b-Nbs from Families A, B, D, G, and I. A nanobody from Family L (which lacks binding to TfR1 on CHEK-ATP089 cells) serves as a negative control. In these experiments, CHEK-ATP089 cells were incubated with the specified proteins for 1 h on ice before FACS analysis. Human Transferrin-FITC and TfR1b-Nbs were utilized at a concentration of 2.5 micromolar

The biological activity of TfR1b-Nbs produced in CHO-S cells was assessed by testing binding to hTfR1. HEK293 cells transfected with hTfR1 and EGFP showed strong staining with TfR1b-Nbs from Families A, B, D, G, and I, but not from Families E, F, J, or L (Supporting Figure S9). Similar results were obtained using CHEK-ATP089, a stable HEK293/hTfR1 cell line, where TfR1 expression was confirmed by transferrin-FITC staining (Fig. 2a).

### Identifying TfR1-Nbs compatible with transferrin-mediated iron transport

To evaluate whether TfR1b-Nbs interfere with TF binding or uptake, we used CHEK-ATP089 cells. As expected, unlabeled TF competes with TF-FITC for receptor binding in a dose-dependent manner—effective at TF/TF-FITC ratios of 3 and 1, but not 0.3—confirming assay sensitivity (Fig. 2b). Using the same setup, we tested whether TfR1b-Nbs (2.5 µM) could reduce TF-FITC binding at a 1:1 molar ratio. All three Family I TfR1b-Nbs reduced TF-FITC binding, while none of the other 11 tested Nbs (from Families A, B, D, and G) showed interference (Fig. 2b).

**Fig. 3:**
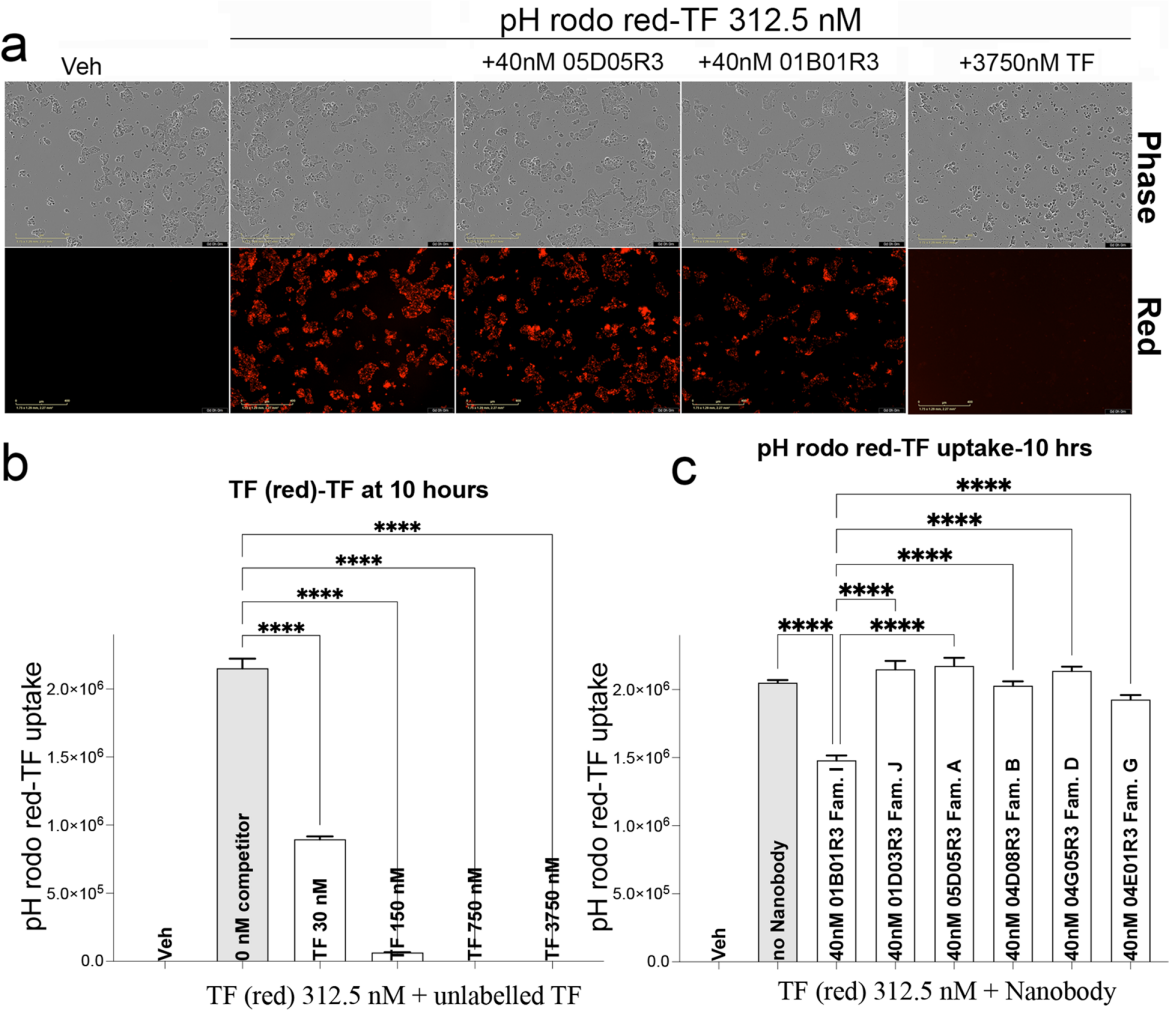
Selection of TfR1b-Nbs that do not interfere with transferrin uptake. **a** Example Incucyte images (phase contrast to visualize cells; red signal shows pHrodo-TF uptake) of vehicle alone, pHrodo-TF at 312.5 nM either alone or with 40 nM of 05D05R3 (Family A), 01B01R3 (Family I) or unlabeled TF at 3750 nM. **b** Competition assay showing dose-dependent inhibition of pHrodo Red–labeled transferrin (TF) uptake by increasing concentrations of unlabeled TF in CHEK-ATP089 cells. Ordinary one-way ANOVA revealed a significant effect of unlabeled TF on uptake (F(5, 66) = 833.5, *p* < 0.0001). Post hoc Šídák's multiple comparisons test showed significant inhibition at 312.5 nM TF (red) compared to 30 nM (*p* < 0.0001), 150 nM (*p* < 0.0001), 750 nM (*p* < 0.0001), and 3750 nM (*p* < 0.0001), confirming robust competitive uptake. **c)** Assessment of the impact of representative TfR1b nanobodies (Nbs) on TF uptake. Nbs from Families A (05D05R3), B (04D08R3), D (04G05R3), G (04E01R3), and I (01B01R3) were tested at 40 nM, along with a Family J nanobody (01D03R3) that does not bind TfR1 on CHEK-ATP089 cells, serving as a negative control. Ordinary one-way ANOVA revealed a significant effect of nanobody treatment (F(7, 88) = 353.9, *p* < 0.0001). Post hoc Šídák's multiple comparisons test indicated that only the Family I Nb (01B01R3) significantly inhibited TF uptake compared to the 312.5 nM TF condition (*p* < 0.0001). In contrast, Nbs from Family A (*p* = 0.3495), Family B (*p* > 0.9999), Family D (*p* = 0.7638), and Family G (*p* = 0.3429) did not show significant inhibition, indicating preserved TF uptake. The Family J Nb (negative control) also had no effect (*p* = 0.6458). Additionally, Family I Nb significantly differed from all other nanobody treatments (*p* < 0.0001 for all comparisons). Data are shown as mean ± SEM of replicate wells from at least three independent experiments. **** *P* < 0.0001

We next assessed whether TfR1b-Nbs affect TF uptake, using pHrodo Red-TF and live-cell imaging on the Incucyte system. pHrodo Red fluorescence increases in acidic compartments, providing a readout of endocytic uptake (Fig. 3a). Unlabeled TF inhibited pHrodo Red-TF uptake in a dose-dependent manner, even at high pHrodo Red-TF/TF ratios (312.5 nM vs. 30 nM) (Fig. 3b), confirm that competition can impair uptake. Consistent with binding data, Family I Nb 01B01R3 significantly reduced pHrodo Red-TF uptake (Fig. 3c), though less potently than 30 nM TF. TfR1b-Nbs from Families A, B, D, and G had no impact (Fig. 3c). A Family J Nb, which does not bind TfR1, also showed no effect (Fig. 3c).

Based on these results, TfR1b-Nbs from Families A, B, D, and G appear most suitable for further development, as they do not interfere with TF-TfR1 interactions and iron uptake.

### Assessing which TfR1b nanobodies cross the BBB in vivo (NewroBus nanobodies)

To test whether TfR1b-Nbs cross the BBB in vivo, we generated knock-in (KI) rats in which the endogenous rat *Tfrc* and *Tf* genes were replaced with their human counterparts. These humanized models were essential becauseTfR1b-Nbs do not bind to rodent TfR1 (see Supporting Figures S5–S8), making conventional rodents unsuitable for evaluating in vivo BBB permeability. By replacing rat *Tfrc* with the human coding sequence (*Tfrc*^*h*^) and rat *Tf* with human *TF* (*Tf*^*h*^), we created a physiologically relevant in vivo platform to assess the BBB transport and safety profile of TfR1-targeted nanobody biologics, as described [13].

**Fig. 4:**
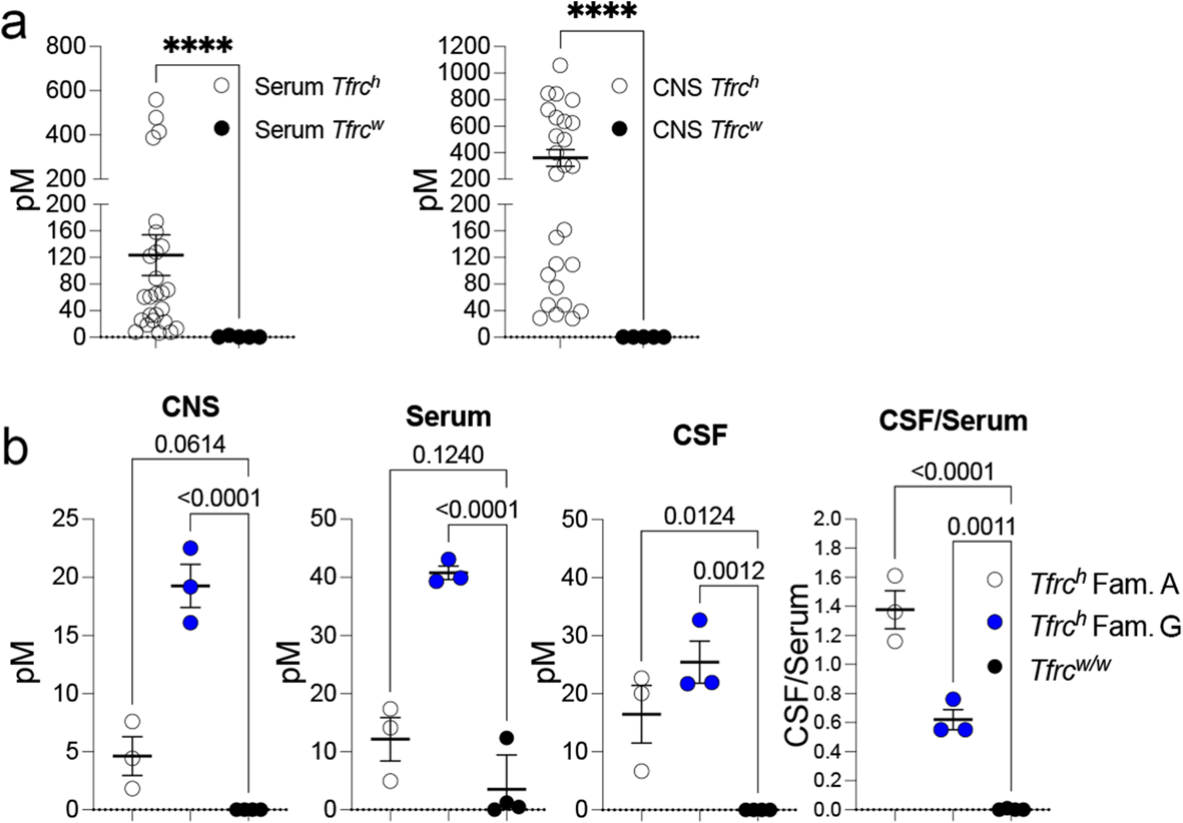
In vivo BBB permeability of TfR1b-Nbs in humanized *Tfrc* knock-in rats. **a** ELISA detection of TfR1b-Nbs in brain homogenates and serum 16–18 h after intravenous injection. Nanobodies were detected in the brains of rats expressing at least one human TfR1 allele (*Tfrc*^*h/w*^ or *Tfrc*^*h/h*^), but not in wild-type controls (*Tfrc*^*w/w*^). Statistical analysis (Mann–Whitney test, a non-parametric method that does not assume normal distribution and is well suited for comparing two independent groups when the data may be skewed or non-normally distributed) revealed significant difference in serum and CSF levels between groups (*p* < 0.0001), with nanobodies found in the CSF of humanized rats compared but not wild-type controls, indicating human TfR1-dependent BBB permeability. **b** Detection of TfR1b-Nbs 04B05R3 (Family A) and 05D01R3 (Family G) in *Tfrc*^*h/w*^ and *Tfrc*^*w/w*^ rats 44–48 h post-injection. In CNS (brain homogenates), ordinary one-way ANOVA revealed a significant effect (*F*(2, 7) = 62.43, *p* < 0.0001). Post hoc Dunnett’s multiple comparisons test showed that 05D01R3 (Family D) was significantly enriched in the CNS of *Tfrc*^*h/w*^ rats compared to wild-type controls (*p* < 0.0001), while 04B05R3 (Family A) showed a non-significant trend (*p* = 0.0614). In serum, ordinary one-way ANOVA showed a significant overall effect (*F*(2, 7) = 44.36, *p* = 0.0001). Dunnett’s test revealed that 05D01R3 nanobody levels were significantly higher in *Tfrc*^*h/w*^ rats compared to wild-type (*p* < 0.0001), whereas Family A levels were not significantly different (*p* = 0.1240). In CSF, a separate ANOVA confirmed a significant genotype effect (*F*(2, 7) = 18.17, *p* = 0.0017). Dunnett’s multiple comparisons test showed significant enrichment for both Family A (*p* = 0.0124) and Family D (*p* = 0.0012) nanobodies in *Tfrc*^*h/w*^ CSF compared to wild-type. CSF/serum ratios also differed significantly between groups (*F*(2, 7) = 86.44, *p* < 0.0001). Dunnett’s test showed that both nanobodies were significantly elevated in *Tfrc*^*h/w*^ rats versus wild-type (*p* < 0.0001 and *p* = 0.0011, respectively), further supporting BBB transcytosis. Data are shown as mean ± SEM

In initial experiments, we used a mixed cohort of *Tfrc*^*h/w*^, *Tfrc*^*h/h*^, and wild-type *Tfrc*^*w/w*^ rats—with additional variability in transferrin humanization levels, including a predominance of *Tf*^*h/w*^ animals, relatively few *Tf*^*h/h*^ and *Tf*^*w/w*^ animals—alongside differences in sex, age, genotype distribution, and tissue collection times—to assess whether TfR1b-Nbs from Families A, B, D, and G can cross the BBB. Due to these variables and the small number of animals injected with each nanobody (summarized in Table 2), the data were pooled to provide an overall estimate of BBB permeability. These pooled results, shown in Fig. 4a, reflect the general ability of TfR1b-Nbs to cross the BBB, but do not allow for direct comparison between individual nanobodies.

**Table 2:** Detection of TfR1b-Nbs in brain samples of rats expressing human TfR1, compared with rat Tfr1-expressing animals. Columns 1–6 list the nanobody name, family, *Tfrc* genotype, sex, age, and time of tissue collection post-injection. Columns 7 and 8 report the concentrations of TfR1b-Nbs in serum and brain homogenates, respectively. Data for this experiment are shown in Fig. 4a

**Nanobody**	**Family**	***Tf***	***Tfrc***	**sex**	**Age in days**	**Time (hrs)**	**Serum [pM]**	**Brain homog. [pM]**
02B02R3	A	*h/w*	*h/h*	f	70	16–18	33.2	28.6
05F02R3	A	*h/h*	*h/h*	f	70	16–18	22.7	28.0
04F01R3	B	*h/w*	*h/h*	m	39	16–18	128.1	241.2
04G05R3	D	*h/w*	*h/h*	m	39	16–18	414.8	624.4
03E01R3	G	*h/w*	*h/h*	m	31	16–18	559.7	843.8
04E01R3	G	*h/w*	*h/h*	f	34	16–18	388.2	796.9
01B01R3	I	*h/w*	*h/h*	m	122	16–18	60.6	109.9
01B01R3	I	*h/w*	*h/h*	f	34	16–18	60.4	299.0
03G10R3	I	*h/w*	*h/h*	m	121	16–18	13.0	48.4
02B02R3	A	*h/w*	*h/w*	m	114	16–18	42.4	94.0
02F06R3	A	*h/w*	*h/w*	m	61	16–18	71.2	161.8
04B05R3	A	*h/w*	*h/w*	f	107	12	478.5	1058.6
04B05R3	A	*h/w*	*h/w*	f	237	16–18	174.1	525.2
05D05R3	A	*h/w*	*h/w*	m	237	16–18	25.0	39.0
05F02R3	A	*h/w*	*h/w*	f	147	16–18	33.4	109.3
04D08R3	B	*h/w*	*h/w*	m	147	16–18	66.7	498.6
04D08R3	B	*w/w*	*h/w*	m	34	16–18	25.4	48.2
04F01R3	B	*h/w*	*h/w*	m	114	16–18	88.5	635.1
04G05R3	D	*h/w*	*h/w*	m	147	16–18	121.9	847.2
03E01R3	G	*h/h*	*h/w*	f	64	16–18	158.3	663.6
04E01R3	G	*h/w*	*h/w*	f	212	16–18	136.6	724.3
05D01R3	G	*w/w*	*h/w*	f	94	12	65.3	398.1
05D01R3	G	*h/w*	*h/w*	m	61	16–18	18.6	306.8
01B01R3	I	*h/w*	*h/w*	m	149	16–18	7.6	74.5
01B06R3	I	*h/h*	*h/w*	m	65	16–18	6.3	34.1
03G10R3	I	*h/w*	*h/w*	m	51	16–18	7.8	150.1
02F06R3	A	*w/w*	*w/w*	m	49	16–18	0.0	0.0
04B05R3	A	*w/w*	*w/w*	f	217	16–18	0.0	0.0
05D05R3	A	*w/w*	*w/w*	m	217	16–18	2.8	0.0
05D01R3	G	*w/w*	*w/w*	m	51	16–18	0.0	0.0
04G05R3	D	*w/w*	*w/w*	f	139	12	0.0	0.0

Rats were injected intravenously with the indicated amounts of nanobodies and processed at defined time points. After blood collection for serum analysis, animals were thoroughly perfused with PBS to remove residual blood from all tissues, including the brain.

All tested TfR1b-Nbs were detected in the brains of *Tfrc*^*h/w*^ and *Tfrc*^*h/h*^ rats, but not in *Tfrc*^*w/w*^ animals, indicating that BBB transcytosis is dependent on human TfR1. Notably, a single human TfR1 allele was sufficient to mediate brain delivery. Serum nanobody levels were also elevated in *Tfrc*^*h/w*^ and *Tfrc*^*h/h*^ rats, perhaps reflecting increased stability in animals expressing the target.

There was no apparent correlation between the degree of Tf humanization and the extent of brain penetration by the nanobodies; therefore, all rats used for BBB permeability tests in the follow-up experiments were wild type for the *Tf* gene, unless otherwise specified. However, because the data were pooled, it was not possible to compare the relative BBB permeability of individual nanobodies.

While the initial pooled dataset suggested that TfR1b-Nbs can cross the BBB via human TfR1, we conducted a follow-up experiment to generate cleaner and more definitive evidence. Nanobodies 04B05R3 (Family A) and 05D01R3 (Family G) were selected for re-testing in a controlled cohort of *Tfrc*^*h/w*^ and wild-type *Tfrc*^*w/w*^ rats. Table 3 provides detailed information on the age, sex, genotype, and nanobody used for each animal. Samples were collected 44–48 hours after intravenous injection and included both serum and cerebrospinal fluid (CSF).

**Table 3:** Detection of TfR1b-Nbs in brain samples of rats expressing human TfR1, compared with rat Tfr1-expressing animals. A human TfR1-specific ELISA detected TfR1b-Nbs in brain tissues and CSF of *Tfr1*^*h/w*^ rats, but not in *Tfr1*^*w/w*^ controls. Columns 1–6 list the nanobody name, family, *Tfr1* genotype, sex, age, and time of tissue collection post-injection. Columns 7–10 report the concentrations of TfR1b-Nbs in serum, CSF, the CSF/serum ratio, and brain homogenates, respectively. Data for this experiment are shown in Fig. 4b

**Nanobody**	**Family**	***Tfrc***	**sex**	**Age in days **	**Time (hrs)**	**Serum [pM] **	**CSF [pM]**	**CSF/Ser.**	**CNS [pM]**
04B05R3	A	*h/w*	m	43	44–48	14.101	22.706	1.61	4.431
04B05R3	A	*h/w*	m	43	44–48	17.371	20.066	1.16	7.593
04B05R3	A	*h/w*	f	42	44–48	4.927	6.6769	1.36	1.83
05D01R3	G	*h/w*	m	44	44–48	43.093	32.691	0.76	22.517
05D01R3	G	*h/w*	m	44	44–48	39.896	21.941	0.55	19.183
05D01R3	G	*h/w*	f	44	44–48	39.326	21.714	0.55	16.104
04B05R3	A	*w/w*	m	42	44–48	1.226	0	0	0
04B05R3	A	*w/w*	m	44	44–48	0.498	0	0	0
05D01R3	G	*w/w*	m	42	44–48	0	0	0	0
05D01R3	G	*w/w*	m	43	44–48	12.371	0	0	0

As shown in Fig. 4b, serum nanobody levels were higher in *Tfrc*^*h/w*^ rats, and only these animals showed nanobody presence in both the brain and CSF. Notably, both nanobodies exhibited high CSF/serum ratios—particularly 04B05R3, which reached ~1.5—suggesting strong and preferential accumulation in the CNS. We report the CSF/serum ratio rather than the CNS/serum ratio because CSF concentrations can be measured directly, whereas CNS tissue concentrations are determined following homogenization, separation, and resuspension steps that introduce variability and distort the true cellular molarity. Moreover, the CSF is continuous with the brain interstitial fluid—the compartment where inflammatory cytokines such as TNFα act on neurons and glia. Since anti-TfR1 nanobodies were originally designed to deliver cytokine-neutralizing nanobodies (e.g., anti-TNFα) to this interstitial space, although they can also be adapted for other classes of therapeutics, CSF concentrations provide a practical and physiologically relevant proxy for drug exposure at the intended site of action.

Remarkably, these nanobodies remained detectable 2 days post-injection. This prolonged persistence in rats expressing human TfR1 is unusual for nanobodies, which generally display short systemic half-lives, and interaction with cell membrane TfR1 could, in principle, enhance clearance through target-mediated drug disposition. Notably, however, we consistently found higher plasma levels of TfR1b-Nbs in humanized TfR1 rats relative to wild-type animals. One possible explanation is that TfR1b-Nbs forms circulating complexes with soluble TfR1 (sTfR1), which may partially protect it from rapid renal elimination, reminiscent of albumin-binding–based half-life extension strategies [14]. While this remains speculative, future pharmacokinetic studies will be essential to clarify the determinants of TfR1b-Nbs half-life and to evaluate potential strategies for half-life extension.

### Assessing CNS delivery of other biologics via TfR1b-Nb-directed transcytosis

**Fig. 5:**
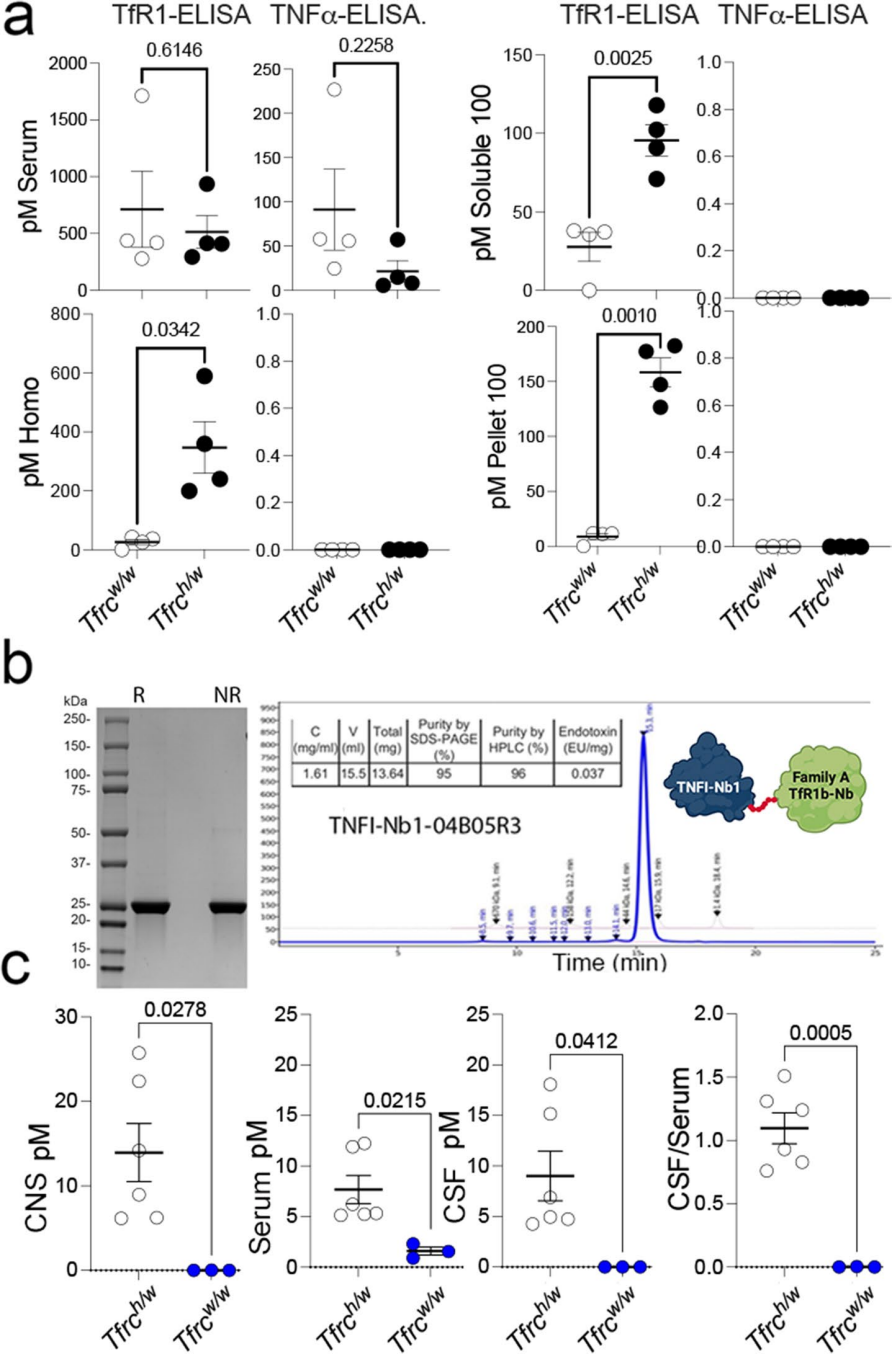
Fusion to TfR1b-Nb enables CNS delivery of TNFI-Nb1 via human TfR1-mediated transcytosis. **a** TNFα-based ELISA quantification of TNFI-Nb1 in serum and brain homogenates from *Tfr1*^*h/w*^ and *Tfr1*^*w/w*^ rats, 6 h after intravenous injection. TNFI-Nb1 was detectable in serum but absent in brain tissue, indicating lack of BBB permeability. **b** SDS-PAGE and SEC-HPLC analysis of the heterodimer composed of TNFI-Nb1 fused to TfR1b-Nb 04B05R3 (Family A), produced in CHO-S cells. **C**) TNFα-based ELISA quantification of TNFI-Nb1 ~ 04B05R3 in serum, CSF, and brain homogenates of *Tfr1*^*h/w*^ and *Tfr1*^*w/w*^ rats, 48 h post-injection. The heterodimer was present in serum of both genotypes but detected in CSF and brain only in *Tfr1*^*h/w*^ rats, demonstrating human TfR1-dependent BBB transcytosis. The observed CSF/serum ratio (TNFα-based ELISA) of ~ 1 indicates efficient CNS delivery. Statistical analyses were conducted using unpaired two-tailed t-tests, with p-values reported in the figure panels. Data are presented as mean ± SEM

We have also produced nanobodies that inhibit TNFα activity, referred to as TNFI-Nbs [15]. We injected *Tfrc*^*w/w*^ and *Tfrc*^*h/w*^ rats with TNFI-Nb1 and TfR1b-Nb 04B05R3 (Family A, see Table 4). The TfR1b-Nb was detectable in the serum of all animals but was predominantly found in the brain homogenates and soluble brain fraction of *Tfrc*^*h/w*^ rats, indicating TfR1-dependent CNS penetration (Fig. 5a). In contrast, TNFI-Nb1 was only detectable in the serum across all animals and was absent from brain tissue, suggesting it does not cross the BBB. Although interpretation is somewhat limited by the apparently faster clearance of TNFI-Nb1, these data overall indicate that TfR1b-Nb crosses the BBB in a human TfR1-dependent manner, while TNFI-Nb1 does not. Importantly, the data also confirm previous data showing that BBB integrity is preserved in *Tfrc*^*h/w*^ rats [13].

**Table 4:** Details of the animals used in the experiment presented in Fig. 5a, including genotype, sex, age, and nanobody administered

**Nanobody**	***Tfrc***	**sex**	**Age in days **	**Time (hrs)**	**Serum [pM] **	**CNS [pM]**
TNFI-Nb1 + TfR1b-Nb 04B05R3	*h/w*	f	48	6	14.81634	0
	*h/w*	f	48	6	5.659766	0
	*h/w*	m	40	6	8.185005	0
	*h/w*	f	108	6	57.38742	0
	*w/w*	f	139	6	226.9179	0
	*w/w*	f	39	6	55.92783	0
	*w/w*	m	39	6	57.97242	0
	*w/w*	m	39	6	24.78752	0

Next, we tested whether fusing TNFI-Nb1 to the TfR1b-Nb 04B05R3 (Family A) could promote brain delivery of TNFI-Nb1. The TNFI-Nb1 and TfR1b-Nb 04B05R3 nanobodies were genetically fused using a flexible glycine-serine–rich linker with the sequence GGGSGGGSGGGSGGG**.** This linker was selected based on two key criteria: 1) It provides structural flexibility while minimizing the risk of steric interference between the two domains; 2) It has low predicted immunogenicity, as it is not expected to generate peptides capable of stimulating T cell responses, thereby reducing the likelihood of anti-drug immune reactions. Production of the heterodimer in CHO-S cells was outsourced to GenScript (Fig. 5b).

As with the monomeric TfR1b-Nbs, heterodimers were administered via IV injection. Animals were extensively perfused with PBS prior to tissue collection to eliminate residual blood from the brain and other organs. Table 5 summarizes the age, sex, genotype of the rats used, and the specific nanobodies injected. While the TfR1b-Nb monomers were detected by ELISA using human TfR1 as the capture reagent, the heterodimers were detected using also an ELISA with human TNFα as the capture moiety [15]. This strategy allowed us to directly evaluate whether a TNFα-binding nanobody can be transported across the BBB when fused to a TfR1b-Nb.

**Table 5:** Shuttle Activity of TfR1b-Nbs for TNFI-Nb1 in brain samples of rats expressing human TfR1. A human TNFα-specific ELISA detected TNFI-Nb1-04B05R3 in brain tissues and CSF of *Tfr1*^*h/w*^ rats, but not in *Tfr1*^*w/w*^ controls. The linker used to link these two nanobodies was: GGGGSGGG. Columns 1–6 list the nanobody name, family, *Tfr1* genotype, sex, age, and time of tissue collection post-injection. Columns 7–10 report the concentrations of TfR1b-Nbs in serum, CSF, the CSF/serum ratio, and brain homogenates, respectively. Data for this experiment are shown in Fig. 5c

**Nanobody**	**Family**	***Tfrc***	**sex**	**Age in days **	**Time (hrs)**	**Serum [pM] **	**CSF[pM]**	**CSF/Serum**	**CNS [pM]**
TNFI-Nb1-04B05R3	A	*h/w*	m	44	44–48	5.14	4.25	0.83	6.23
TNFI-Nb1-04B05R3	A	*h/w*	f	44	44–48	5.32	4.95	0.93	6.17
TNFI-Nb1-04B05R3	A	*h/w*	m	44	44–48	6.21	4.73	0.76	8.96
TNFI-Nb1-04B05R3	A	*h/w*	f	51	44–48	5.24	6.88	1.31	14.18
TNFI-Nb1-04B05R3	A	*h/w*	m	50	44–48	12.23	15.16	1.24	25.73
TNFI-Nb1-04B05R3	A	*h/w*	m	51	44–48	11.91	18.08	1.51	22.40
TNFI-Nb1-04B05R3	A	*w/w*	m	51	44–48	0.91	0.004	0.004	0.00
TNFI-Nb1-04B05R3	A	*w/w*	f	44	44–48	2.30	0.004	0.001	0.03
TNFI-Nb1-04B05R3	A	*w/w*	f	44	44–48	1.56	0.002	0.001	0.00

The heterodimer was detected in both the brain and CSF of *Tfrc*^*h/w*^ rats, but not in wild-type *Tfrc*^*w/w*^ controls, confirming human TfR1-dependent BBB transport (Fig. 5c). Similar to the monomeric TfR1b-Nb 04B05R3, the heterodimer showed strong CNS localization, with a CSF/serum ratio of approximately 1—slightly lower than the monomer (~1.5), but still indicative of efficient brain accumulation.

To further evaluate BBB transcytosis of the heterodimer, we applied a fractionation protocol [16] used by Denali to assess CNS penetration of AVI-based therapeutics. One hemibrain from each of three *Tfrc*^*h/w*^ rats was separated into vasculature and vascular-depleted parenchymal fractions. In this experiment, we tested the TNFI-Nb1–linker–04B05R3 heterodimer. The goal of this experiment is to generate a parenchymal fraction depleted of vasculature. This is essential to determine whether the nanobody signal detected in whole brain homogenates is simply due to binding of TfR1b-Nbs to endothelial human TfR1 in blood vessels (and possibly internalized via endocytosis) or instead reflected penetration into parenchymal brain cells.

**Fig. 6:**
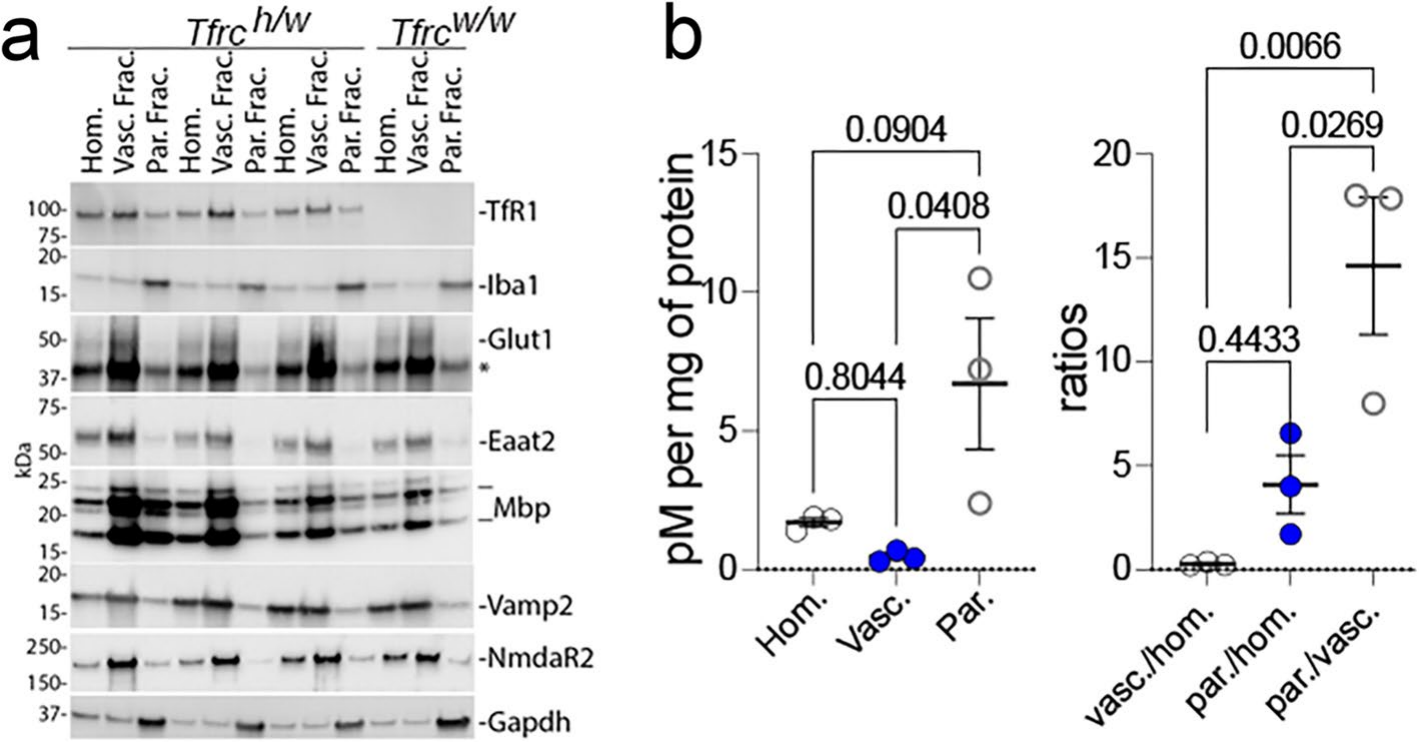
Brain fractionation analysis of TNFI-Nb1–linker–04B05R3 distribution in Tfr1^h/w^ rats. **a** Western blot validation of brain fractionation into vasculature and parenchyma. Glut1 (endothelial marker) was enriched in the vasculature fraction, while Gapdh and cell-type–specific markers—Vamp2 and NmdaR2b (neurons), Iba1 (microglia), Eaat2 (astrocytes), and Mbp (oligodendrocytes)—were enriched in the parenchymal fraction. Human TfR1 was detected in all fractions, with strongest signal in the vasculature. **b** ELISA quantification of TNFI-Nb1–04B05R3 in homogenate, vasculature, and parenchymal fractions. After normalization to protein content, the parenchymal fraction showed the highest nanobody levels, supporting uptake by CNS cells via human TfR1-mediated transcytosis. Ordinary one-way ANOVA showed a significant overall effect (F(2, 6) = 5.871, *p* = 0.0387). Post hoc Tukey's multiple comparisons test showed significant differences in the parenchymal fractions, as indicated in the figure. Analysis of the ratio by ordinary one-way ANOVA showed a significant overall effect (F(2, 6) = 12.81, *p* = 0.0068). Post hoc Tukey's multiple comparisons test confirmed a significant relative increase in TNFI-Nb1–04B05R3 in parenchymal fractions compared to both homogenate and vasculature fractions. The p-values are indicated in the figure

Western blot analysis confirmed effective fractionation; the endothelial marker Glut1 was enriched in the vasculature fraction and depleted in the parenchyma, while Gapdh—a general cellular marker—was enriched in the parenchyma. Cell-type–specific markers in the parenchymal fraction included: Vamp2 (neurons), NmdaR2b (neurons), Iba1(microglia), Eaat2 (astrocytes), and Mbp (oligodendrocytes) (Fig. 6a). These results indicate successful enrichment of parenchymal CNS cell types, with particularly strong signal for microglial marker. Human TfR1 was detected in all brain fractions of *Tfrc*^*h/w*^ rats, with highest expression in the vasculature, consistent with its known highest endothelial localization.

ELISA analysis (Fig. 6b) confirmed the presence of TNFI-Nb1–04B05R3 in brain homogenates, vasculature, and parenchymal fractions of *Tfrc*^*h/w*^ rats. After normalization to protein content, the parenchymal fraction showed the highest levels, supporting uptake of the heterodimer by CNS cells, likely via human TfR1.

These results support the concept that TfR1b-Nbs can serve as effective "NewroBuses," delivering otherwise BBB-impermeable biologics into the brain.

### Assessment of NewroBus-directed brain uptake of a non–BBB-penetrant biologic

**Fig. 7:**
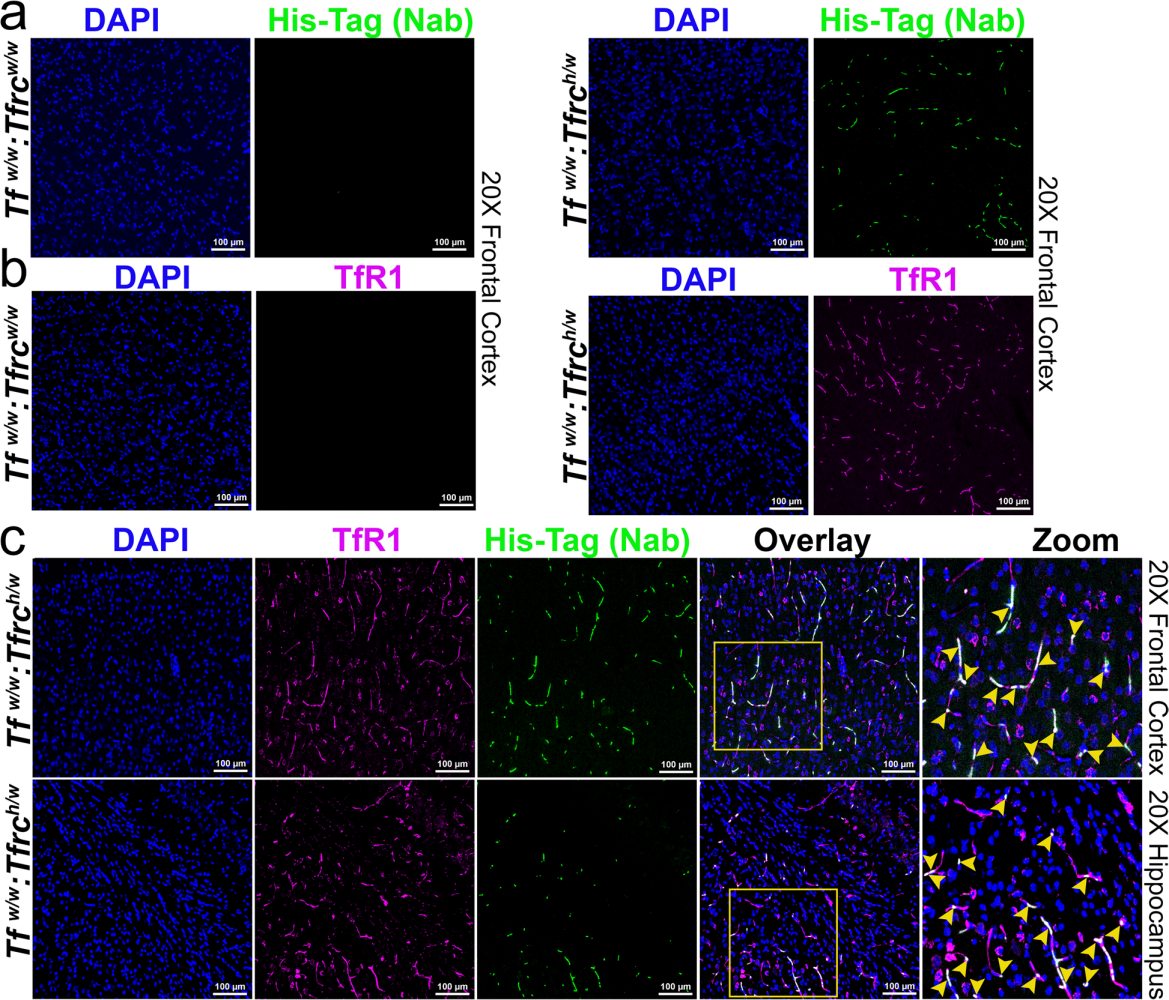
TNFI-Nb1-linker-04B05R3 colocalize with human TfR1 in brain vessels of *Tfr1*^*h/w*^ rats**. A** Anti-His tag antibody (green) and (**B**) anti-human TfR1 antibody (magenta) stain TNFI-Nb1-linker-04B05R3 and human TfR1, respectively, in the brains of *Tfr1*^*h/w*^ but not *Tfr1*^*w/w*^ rats. **C**) Human TfR1 (magenta), and TNFI-Nb1-linker-04B05R3 (green) colocalize in vessels in the brains of *Tfr1*^*h/w*^ rats. Yellow arrows in the zoomed-in panel indicate the colocalization spots within the region highlighted by the yellow square in the overlay panel

To confirm human TfR1-dependent BBB penetration of TNFI-Nb1-04B05R3, we performed IHC. One hemibrain from each rat used in the experiment shown in Fig. 6 was fixed in 4% paraformaldehyde following perfusion. Brain sections from *Tfrc*^*h/w*^ and *Tfrc*^*w/w*^ rats were stained using anti-His tag (for the nanobody) and anti-human TfR1 antibodies to examine nanobody localization and target engagement. TNFI-Nb1–04B05R3 (green) was detected in *Tfrc*^*h/w*^ but not *Tfrc*^*w/w*^ brains (Fig. 7a). Figure 7b confirms human TfR1 expression (magenta) exclusively in *Tfrc*^*h/w*^ brains. Colocalization of TNFI-Nb1–04B05R3 (green) and human TfR1 (magenta)was observed in *Tfrc*^*h/w*^ rats (Fig. 7c). Merged and zoomed-in images show clear colocalization, confirming binding to the brain endothelium in a TfR1-dependent manner.

**Fig. 8:**
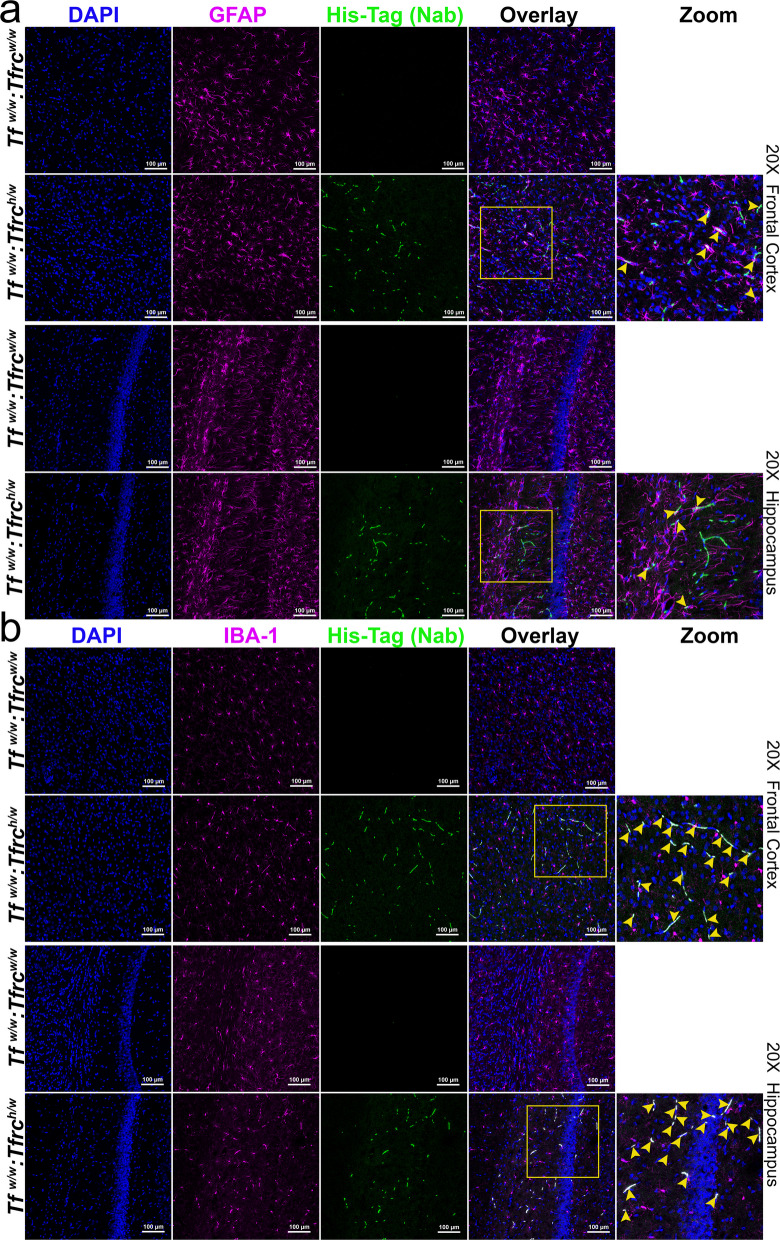
Confocal images showing the localization of TNFI-Nb1-linker-04B05R3 in astrocytes and microglia of the cortex and hippocampus in *Tfr1*^*h/w*^ and *Tfr1*^*w/w*^ rats. **a** Sections were stained with an anti-His tag antibody (green) to detect TNFI-Nb1-linker-04B05R3 and an anti-GFAP antibody (magenta) to label astrocytes. TNFI-Nb1-linker-04B05R3 is present in the brains of *Tfr1*^*h/w*^ rats but not in *Tfr1*^*w/w*^ rats. In *Tfr1*^*h/w*^ rats, TNFI-Nb1-linker-04B05R3 colocalizes with some astrocytes, indicated by yellow overlays in the merged images. White arrows in the zoomed-in panel point to colocalization spots within the region highlighted by the white square in the overlay panel. **b** Sections were stained with an anti-His tag antibody (green) to detect TNFI-Nb1-linker-04B05R3 and an anti-IBA1 antibody (magenta) to label microglia. TNFI-Nb1-linker-04B05R3 is present in the brains of *Tfr1*^*h/w*^ rats but not in *Tfr1*^*w/w*^ rats. In *Tfr1*^*h/w*^ rats, TNFI-Nb1-linker-04B05R3 colocalizes with most microglia, as indicated by yellow overlays in the merged images. Yellow arrows in the zoomed-in panel point to colocalization spots within the region highlighted by the white square in the overlay panel

The presence of the heterodimer in the parenchymal fraction suggests cellular uptake within the CNS. To investigate whether TNFI-Nb1–04B05R3 localizes to specific brain cell types, we performed confocal imaging of cortex and hippocampus sections stained with DAPI (nuclei), GFAP (astrocytes), IBA1 (microglia), and an anti-His tag antibody to detect TNFI-Nb1–04B05R3. TNFI-Nb1–04B05R3 colocalizes with GFAP-positive astrocytes (white signal) in both cortex and hippocampus of *Tfrc*^*h/w*^ rats, but not in *Tfrc*^*w/w*^ controls (Fig. 8a). Figure 8b shows similar colocalization with IBA1-positive microglia, again only in *Tfrc*^*h/w*^ rats.

Together, these data demonstrate that TNFI-Nb1–04B05R3 crosses the BBB in a human TfR1-dependent manner, localizing to the CSF—an accessible surrogate for interstitial fluid within the brain parenchyma—with a portion taken up by CNS cells, predominantly astrocytes and microglia. The absence of signal in *Tfrc*^*w/w*^ rats underscores the requirement for human TfR1. These findings support the potential of TfR1b-Nbs as targeted delivery vehicles for otherwise BBB-impermeable therapeutics.

### In silico humanization and developability optimization of lead TfR1b nanobodies

Humanization and optimization are critical steps to enhance nanobody stability, solubility, and expression, while minimizing immunogenicity—key for safe and effective therapeutic use in humans. Accordingly, TfR1b-Nbs from Families A, B, D, and G underwent in silico humanization and developability optimization using a strategy similar to that applied to TNFI-Nbs [15]. We employed AbNatiV, a machine learning algorithm that predicts both humanness and VHH-nativeness directly from sequence [17]. Humanness scores above 0.8 indicate high similarity to human variable domains and correlate with lower immunogenicity risk, while scores below 0.8 suggest greater likelihood of immune recognition due to non-human sequence features.

Based on the most favorable starting points for humanness, VHH-nativeness, and CamSol solubility [18], TFRI1b-Nbs 05F02, 04F01, and 04G05 were selected for TNF1b-Nbs Families A, B, and D, respectively. For Family G, 03E01 and 05D01 were chosen as representatives of the two subfamilies present in this group (Table 6).Two complementary mutational sampling strategies were applied: enhanced sampling, which rapidly converges on an optimized humanized sequence, and exhaustive sampling, which systematically evaluates all permissible residue substitutions based on position-specific scoring matrices (PSSMs) from human VH and VHH databases. Both methods identify designs on the Pareto front, balancing increased humanness with preserved VHH-nativeness—critical for maintaining nanobody stability and folding in the absence of a VL domain.

**Table 6:** AbNatiV Assessment of TfR1b-Nbs. Key metrics include Humaness (H), VHH-ness (VH), and CamSol Intrinsic (S). The Nbs underlined, in bold and italic have been selected for humanization

**ID**	**F**	**H**	**V**	**S**
02F06	**A**	0.721	0.744	0.382
05D05		0.697	0.783	0.405
***05F02***		0.754	0.787	0.357
02B02		0.745	0.785	0.291
04B05		0.734	0.772	0.304
04D08	**B**	0.774	0.831	0.620
***04F01***		0.763	0.847	0.628
***04G05***	**D**	0.763	0.850	0.438
***05D01***	**G**	0.634	0.732	0.505
***03E01***		0.619	0.719	0.042
04E01		0.601	0.735	0.026

Humanization was restricted to solvent-exposed framework residues, while CDRs were preserved to avoid compromising antigen binding. As a further control for structural integrity post-humanization, the structures of wild-type (WT) and all humanized sequences were modelled with NanobodyBuilder 2 [19]. The modelled structures were superimposed based on their framework regions, and the root-mean-square deviation (RMSD) calculated for the CDR regions was approximately 1Å. This value is significantly smaller than the expected modelling accuracy for these regions suggesting minimal or no displacement of the CDR loops resulting from the humanizing framework mutations.

Following in silico humanization, we used the structural models of both Enhanced and Exhaustive humanized variants as inputs for the CamSol Combination pipeline [20], by excluding all CDR regions from the design and by using an alignment of human VH sequences as input, rather than the default VHH sequences. CamSol Combination automatically identifies combinations of mutations predicted to improve solubility and conformational stability, or one of these properties without affecting the other. As a further computational filter, the apparent melting temperature of in silico mutants was predicted with NanoMelt [21]. Of the mutations suggested by CamSol Combination, we retained only those that didn’t reduce humanness according to AbNatiV scoring (Table 7). These variants were produced in CHO-S cells with production performed at GenScript.

**Table 7:** TfR1b-Nb mutants with enhanced therapeutic potential determined by AbNatiV analysis. Changes in Humanness (H), VHH-ness (VH), and CamSol Intrinsic (S) are presented in the last three columns

**ID**	**H**	**V**	**S**	**Code**	**Fam.**
**05F02**	0.754	0.787	0.357		**A**
Enhanced WT-CDR3STEMS	0.790	0.751	0.405		
enhancedWT-CDR3STEM-W	0.828	0.768	0.299	**A2**	
enhanced	0.841	0.782	0.504	**A3**	
Enhanced WT-CDR3STEM-W v1	0.821	0.769	0.598		
enhanced v3	0.840	0.753	0.602		
exhaustive_7	0.868	0.812	0.441	**A4**	
enhanced v1	0.812	0.749	0.800		
enhanced v2	0.827	0.769	0.802		
**04F01**	0.763	0.847	0.628		**B**
Enhanced WT-CDR3STEM	0.870	0.869	0.828	**B1**	
enhanced	0.882	0.866	0.960		
Enhanced WT-CDR3STEM v1	0.867	0.878	0.823	**B2**	
enhanced v1	0.884	0.850	1.029		
exhaustive_12	0.899	0.876	0.978		
enhanced v2	0.882	0.869	0.991		
enhanced v3	0.884	0.858	1.023		
**04G05**	0.763	0.850	0.438		**D**
Enhanced WT-CDR3Stem	0.846	0.860	0.632	**D1**	
enhanced	0.854	0.863	0.847		
Enhanced WT-CDR3Stem v1	0.834	0.854	0.667	**D2**	
exhaustive	0.843	0.841	0.572	**D3**	
enhanced v1	0.814	0.838	0.939		
enhanced v2	0.831	0.854	0.888		
**05D01**	0.763	0.850	0.438		**G**
enhanced_QAP_insertion	0.802	0.841	0.641		
enhanced_QAP_insertion v1	0.796	0.833	0.711		
**03E01**	0.619	0.719	0.042		
exhaustive QAP_insertion	0.793	0.817	0.584		
enhanced	0.784	0.820	0.500		

CamSol Combination automatically identifies combinations of mutations predicted to improve solubility and conformational stability, or one of these properties without affecting the other. The apparent melting temperature of in silico mutants was predicted with NanoMelt [20]. Of the mutations suggested by this approach, we retained only those that didn’t reduce humanness according to AbNatiV scoring (Table 7). These variants were produced in CHO-S cells with production performed at GenScript.

**Fig. 9:**
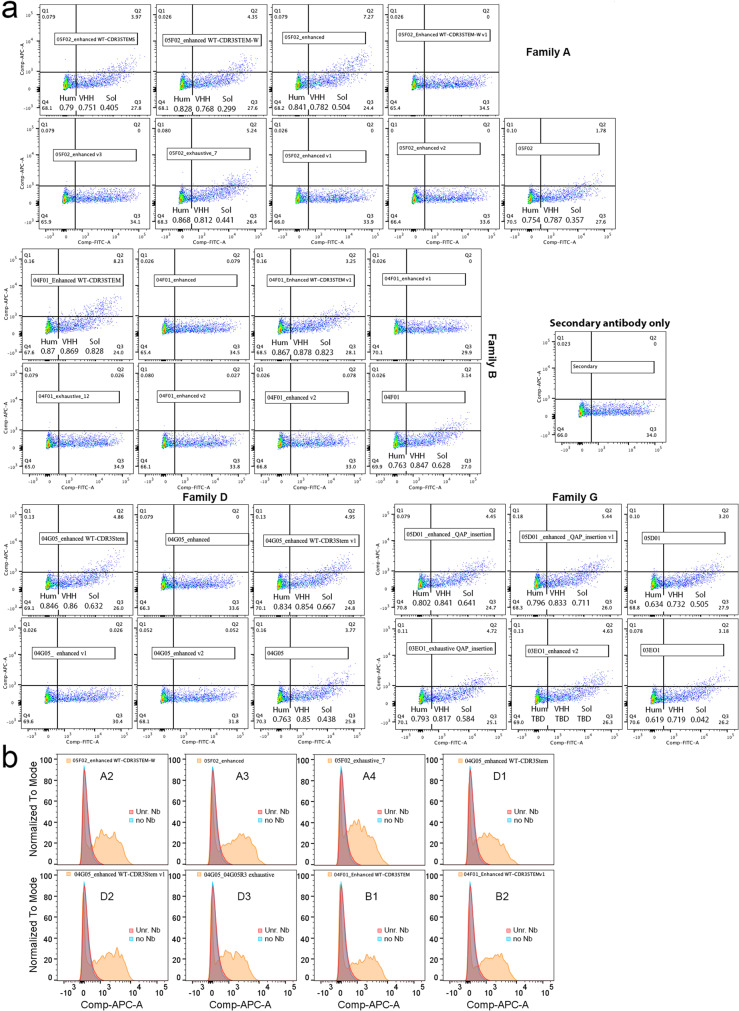
Binding of humanized mutant TfR1b-Nbs to human TfR1. **a** HEK293 cells were transfected with a vector expressing human TfR1 alongside EGFP. The cells were then treated with each TfR1b-Nb at a concentration of 400 nM, followed by incubation with an anti-His-APC antibody. Secondary antibody staining alone is shown as a negative control. Binding of the wild-type TfR1-Nb from which the mutants originated is also shown for comparison. **b** HEK293 cells stably expressing human TfR1 were incubated with the eight indicated nanobody mutants (also known as A2, A3, A4, D1, D2, D3, B1 and B2). Two negative controls were included: (1) secondary antibody only (no nanobody), and (2) a non–TfR1-binding nanobody (Unr. Nb). All eight mutants showed robust binding to human TfR1, as compared to the negative controls

We evaluated these humanized mutants for binding to TfR1 on the cell surface of transiently transfected HEK cells (Fig. 9a) and found that 13 optimized designs retained binding to cell-membrane TfR1. However, one humanized mutant sample was missing from this experiment—the exhaustive Family D mutant. In particular, all designs that lost binding originated from WT nanobodies that had very unusual residues in the STEM of their CDR3 loop according to the AHo numbering scheme. Such residues were spotted as potential liabilities by the algorithms, and mutations were suggested at their sites. However, we reasoned that the presence of these unusual residues in the WT nanobodies was suggestive of an important role for target engagement, and hence we also made some designs that retained such WT residues (denoted as “WT-CDR3STEM” in Table 7). Six out of 7 of these designs retained binding (Fig. 9a).

To confirm whether the transiently transfected mutants truly bound human TfR1, we tested seven mutants with optimal drug development properties—including high predicted solubility and humanness (see Table 7; mutants A2, A3, A4, B1, B2, D1, and D2)—as well as the Family D exhaustive mutant (renamed D3 in Table 7), which had been lost in the previous experiment. All eight mutants were tested on HEK293 cells stably expressing human TfR1. As shown in Fig. 9B, all bound human TfR1 efficiently.

**Fig. 10:**
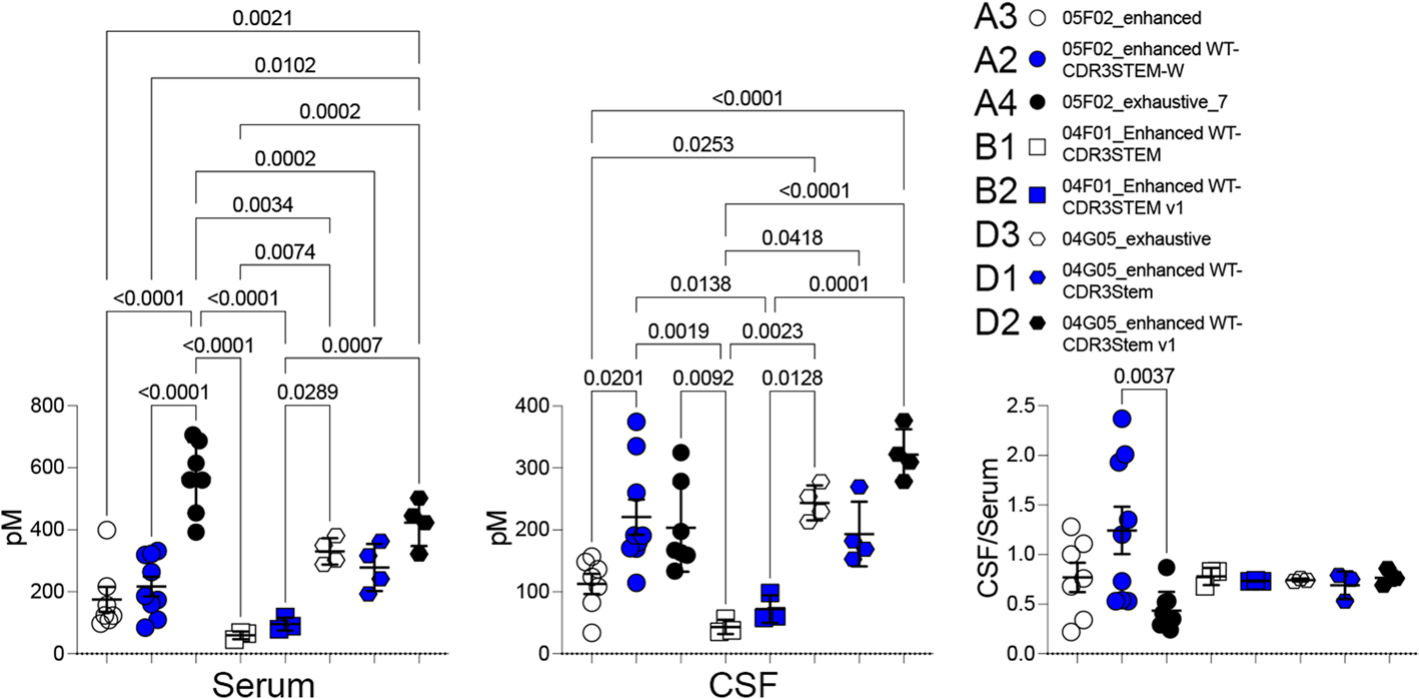
In vivo BBB permeability of eight optimized TfR1b-Nbs in *Tfrc*^*h/w*^ rats. All eight TfR1b nanobody variants showed detectable CSF levels 16–18 h post-injection in *Tfrc*^*h/w*^ rats, confirming successful blood–brain barrier (BBB) penetration via human TfR1. Among these, 05F02_enhanced WT-CDR3STEM-W demonstrated the highest CSF/serum ratio, suggesting optimal CNS transcytosis and stability. Animal genotypes, nanobody sequences, and dosing details are provided in Table 9. In serum, ordinary one-way ANOVA revealed a significant overall effect (F(7, 33) = 18.71, *p* < 0.0001). Tukey’s multiple comparisons test showed that 05F02_exhaustive_7 had significantly lower serum levels than 05F02_enhanced (adjusted *p* < 0.0001), and 04G05_enhanced WT-CDR3STEM v1 also showed significantly lower levels than both 05F02_enhanced (*p* = 0.0021) and 05F02_enhanced WT-CDR3STEM-W (*p* = 0.0102). All other pairwise differences were not statistically significant. In CSF, one-way ANOVA indicated a significant overall difference (F(7, 33) = 9.619, *p* < 0.0001). Tukey’s test revealed that 05F02_enhanced WT-CDR3STEM-W had significantly higher CSF levels than 05F02_enhanced (*p* = 0.0201), 04F01_Enhanced WT-CDR3STEM (*p* = 0.0019), and 04F01_Enhanced WT-CDR3STEM v1 (*p* = 0.0138). Additionally, 04F01_Enhanced WT-CDR3STEM had significantly reduced CSF accumulation compared to 04G05_enhanced WT-CDR3STEM v1 (*p* < 0.0001), and 04F01_Enhanced WT-CDR3STEM v1 also showed lower levels than 04G05_enhanced WT-CDR3STEM v1 (*p* = 0.0001). Other comparisons were not statistically significant. For CSF/serum ratios, ordinary one-way ANOVA also showed a significant effect (F(7, 33) = 2.659, *p* = 0.0269). The only statistically significant difference was observed between 05F02_enhanced WT-CDR3STEM-W and 05F02_exhaustive_7 (adjusted *p* = 0.0037), with the former showing a higher CSF/serum ratio. All other comparisons, including those between 05F02_enhanced and the remaining variants, did not reach significance (adjusted *p* values > 0.3 to > 0.9999). Data are presented as mean ± SEM

Consequently, we assessed their BBB permeability in vivo using our *Tfrc* humanized rats. All eight TfR1b-Nbs exhibited high CSF levels, indicating human TfR1-dependent BBB transcytosis (Fig. 10). Among them, 05F02_enhanced WT-CDR3STEM-W demonstrated the highest CSF/serum ratio. Table 8 provides details on the age, sex, genotype of the rats used, and the specific nanobodies administered.

**Table 8:** Assessment of BBB permeability of optimized TfR1b-Nbs

**Nanobody**	**Family**	***Tfrc***	**sex**	**Age in days**	**Time (hrs)**	**Conc. in Serum [pM]**	**Conc. in CSF [pM]**	**CSF/Ser.**
05F02_enhanced	A	*h/h*	m	131	16–24	122.75	156.99	1.28
05F02_enhanced	A	*h/h*	m	131	16–24	216.82	148.09	0.68
05F02_enhanced	A	*h/h*	m	100	16–24	123.96	124.15	1.00
05F02_enhanced	A	*h/h*	m	100	16–24	97.37	108.01	1.11
05F02_enhanced	A	*h/w*	m	212	16–24	399.12	136.37	0.34
05F02_enhanced	A	*h/w*	f	212	16–24	156.72	33.77	0.22
05F02_enhanced	A	*h/w*	f	212	16–24	108.90	82.48	0.76
05F02_enhanced WT-CDR3STEM-W	A	*h/h*	m	131	16–24	319.32	169.85	0.53
05F02_enhanced WT-CDR3STEM-W	A	*h/h*	m	131	16–24	331.44	179.84	0.54
05F02_enhanced WT-CDR3STEM-W	A	*h/h*	m	131	16–24	322.09	170.56	0.53
05F02_enhanced WT-CDR3STEM-W	A	*h/h*	m	100	16–24	263.54	191.09	0.73
05F02_enhanced WT-CDR3STEM-W	A	*h/w*	m	82	16–24	109.81	260.22	2.37
05F02_enhanced WT-CDR3STEM-W	A	*h/w*	f	94	16–24	173.53	335.17	1.93
05F02_enhanced WT-CDR3STEM-W	A	*h/w*	f	96	16–24	159.62	190.97	1.20
05F02_enhanced WT-CDR3STEM-W	A	*h/w*	f	94	16–24	186.12	374.53	2.01
05F02_enhanced WT-CDR3STEM-W	A	*h/w*	m	82	16–24	84.60	114.36	1.35
05F02_exhaustive_7	A	*h/h*	m	131	16–24	615.23	324.83	0.53
05F02_exhaustive_7	A	*h/h*	m	100	16–24	560.37	197.48	0.35
05F02_exhaustive_7	A	*h/h*	m	131	16–24	687.31	278.54	0.41
05F02_exhaustive_7	A	*h/h*	m	100	16–24	392.66	133.47	0.34
05F02_exhaustive_7	A	*h/w*	m	87	16–24	561.22	163.28	0.29
05F02_exhaustive_7	A	*h/w*	f	212	16–24	454.19	159.10	0.35
05F02_exhaustive_7	A	*h/w*	f	225	16–24	706.45	167.02	0.24
04F01_Enhanced WT-CDR3STEM	B	*h/w*	m	92	16–24	64.86	56.40	0.87
04F01_Enhanced WT-CDR3STEM	B	*h/w*	f	92	16–24	68.06	35.65	0.52
04F01_Enhanced WT-CDR3STEM	B	*h/w*	f	90	16–24	45.49	37.75	0.83
04F01_Enhanced WT-CDR3STEM v1	B	*h/w*	m	92	16–24	118.29	98.00	0.83
04F01_Enhanced WT-CDR3STEM v1	B	*h/w*	f	92	16–24	89.13	60.64	0.68
04F01_Enhanced WT-CDR3STEM v1	B	*h/w*	f	90	16–24	79.08	57.87	0.73
04G05_exhaustive	D	*h/w*	m	84	16–24	381.19	277.88	0.73
04G05_ exhaustive	D	*h/w*	f	89	16–24	288.57	213.07	0.74
04G05_exhaustive	D	*h/w*	m	89	16–24	302.11	229.89	0.76
04G05_ exhaustive	D	*h/w*	f	91	16–24	349.42	253.78	0.73
04G05_enhanced WT-CDR3Stem	D	*h/w*	m	82	16–24	362.82	269.43	0.74
04G05_enhanced WT-CDR3Stem	D	*h/w*	f	91	16–24	316.27	168.69	0.53
04G05_enhanced WT-CDR3Stem	D	*h/w*	m	89	16–24	241.76	182.20	0.75
04G05_enhanced WT-CDR3Stem	D	*h/w*	f	89	16–24	192.70	152.89	0.79
04G05_enhanced WT-CDR3Stem v1	D	*h/w*	m	84	16–24	321.78	278.02	0.86
04G05_enhanced WT-CDR3Stem v1	D	*h/w*	f	94	16–24	445.31	309.46	0.69
04G05_enhanced WT-CDR3Stem v1	D	*h/w*	f	94	16–24	423.02	322.14	0.76
04G05_enhanced WT-CDR3Stem v1	D	*h/w*	f	96	16–24	502.33	376.44	0.75

**Fig. 11:**
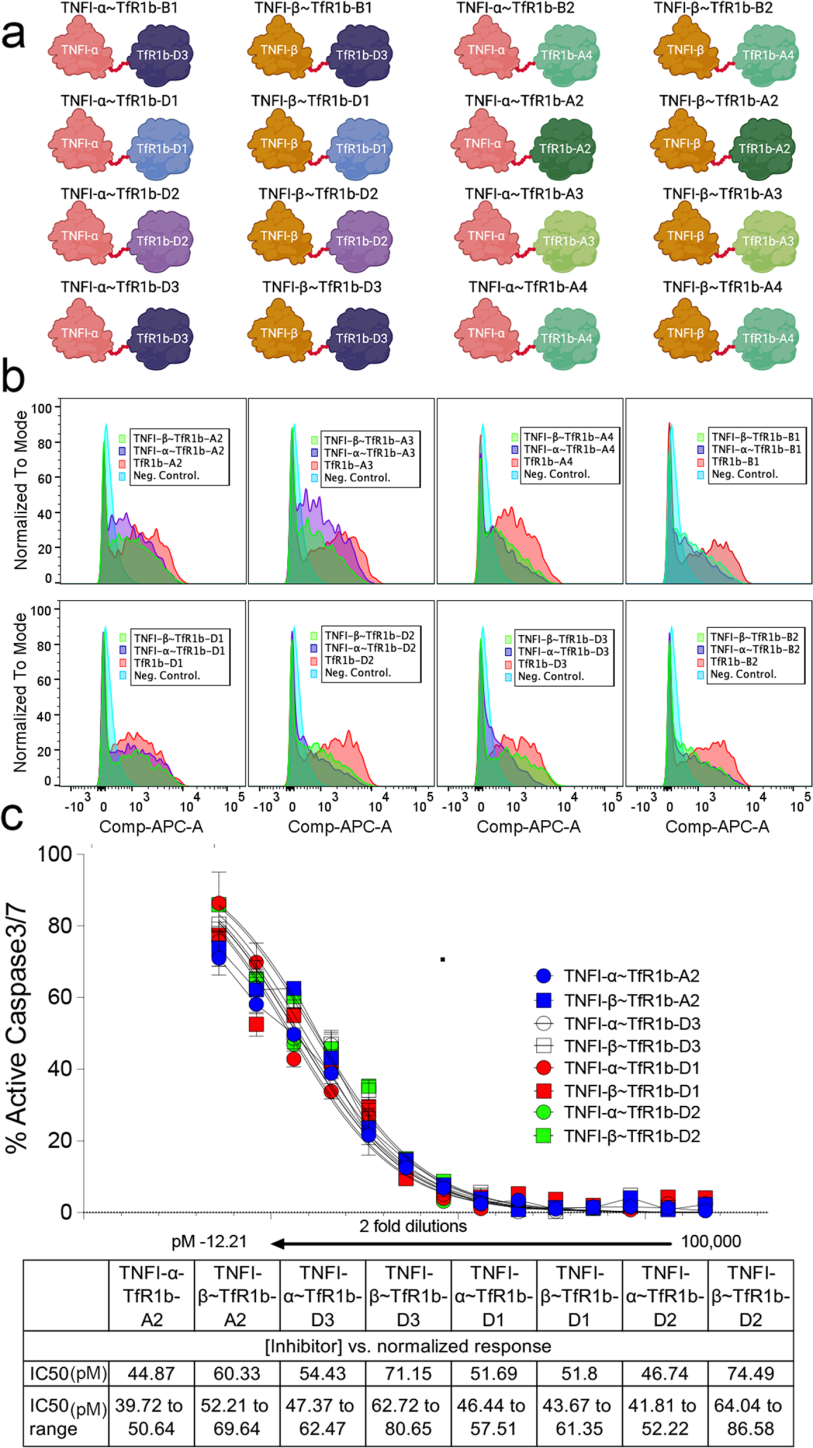
TfR1 binding and TNFα inhibitory activity of optimized and humanized heterodimers. **a** Schematic representation of the 16 humanized and optimized TNFI–TfR1b heterodimers. **b** Flow cytometry analysis in HEK293 cells stably expressing human TfR1, comparing the binding of heterodimers to that of their parental humanized TfR1b-Nbs. **c**) Dose–response apoptosis inhibition curves in WEHI-13VAR cells treated with human TNFα and twofold serial dilutions of TNFI–TfR1b heterodimers (100,000 to 12.21 pM). Apoptosis was measured using a fluorogenic caspase-3/7 activation assay. Caspase-3/7 activity in the TNFα-only samples was defined as 100%. The IC₅₀ values of the heterodimers were comparable to those of the parental humanized nanobodies TNFI-α (IC₅₀ = 48.84 pM, range: 42.11–56.56) and TNFI-β (IC₅₀ = 64.31 pM, range: 57.17–72.31).). Data are presented as mean ± SEM from triplicate measurements and were analyzed using the "Inhibitor vs. Normalized Response" model in GraphPad Prism 10 software

The eight optimized TfR1b-Nbs were fused to either TNFI-α or TNFI-β—two humanized TNFα inhibitors derived from TNFI-Nb1 [15]—to generate 16 unique heterodimers (Fig. 11a). We first evaluated the ability of these heterodimers to bind cell-surface human TfR1, comparing their binding profiles to those of the corresponding parental humanized TfR1b-Nbs used in their construction. Several heterodimers, particularly those incorporating TfR1b-A4, -B1, and -B2, seem to exhibit reduced binding to human TfR1 (Fig. 11b), and further experiments will be required to quantify and confirm this observation.

For the purposes of this study, we prioritized heterodimers containing TfR1b-D1, -D2, -D3, and -A2 for further evaluation. Notably, all selected heterodimers retained TNFα inhibitory activity comparable to TNFI-α and TNFI-β (Fig. 11c) [15]—supporting their continued development as therapeutic candidates.

### Evaluating the potential of subcutaneous administration

**Fig. 12:**
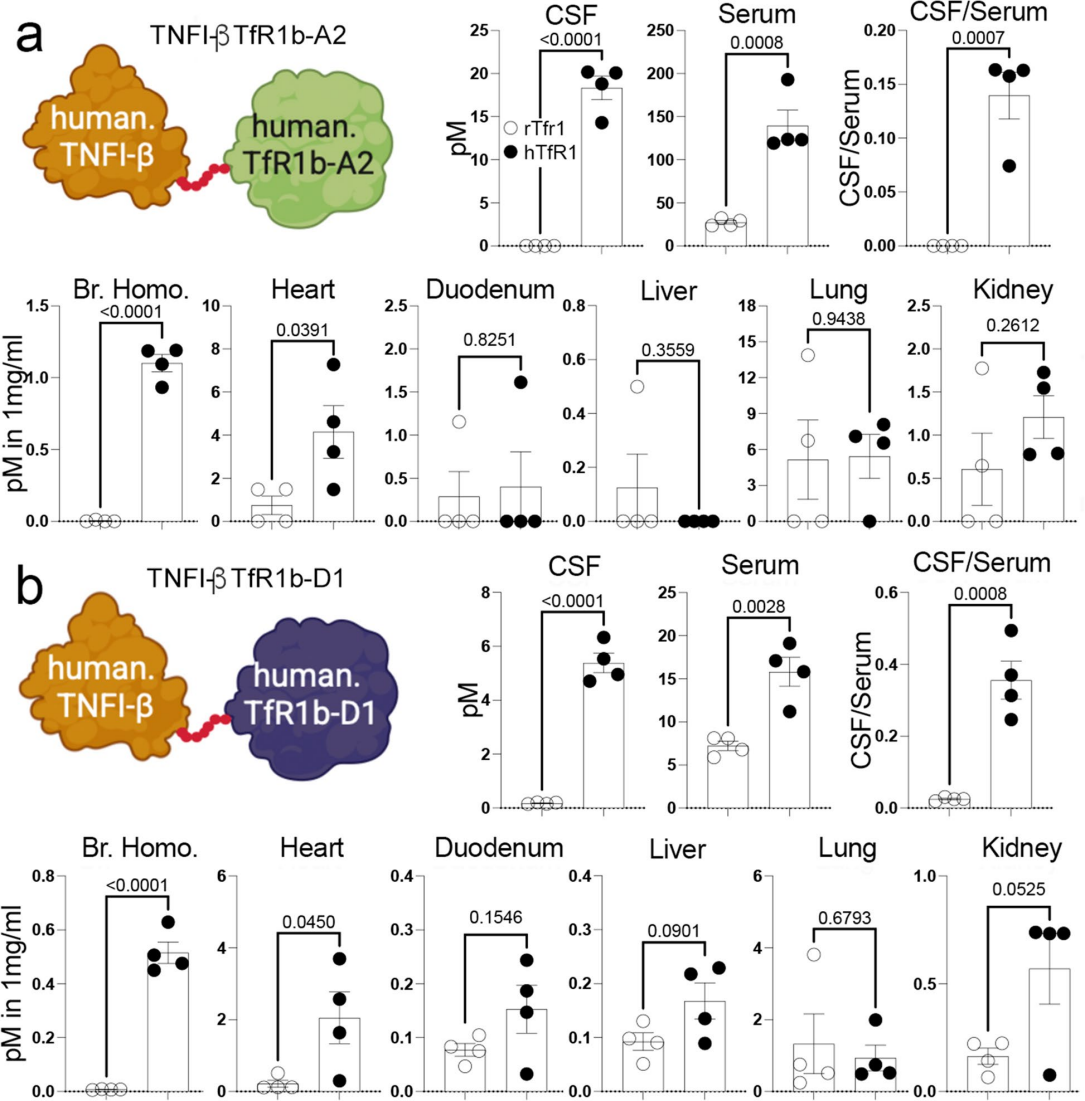
Subcutaneous delivery of humanized heterodimers demonstrates effective BBB transcytosis and extended in vivo stability. *Tfrc*^*h/w*^ and *Tfrc*^*w/w*^ rats were injected subcutaneously with TNFI-β–TfR1b-A2 (**a**) and TNFI-β–TfR1b-D1 (**b**). Heterodimers levels were measured 72 h post-injection. TNFI-β–TfR1b-A2 achieved higher serum and CSF levels (CSF/serum ratio: 0.14), while TNFI-β–TfR1b-D1 showed superior BBB permeability (CSF/serum ratio: 0.37). Heterodimers remained detectable in both CSF and serum three days post-injection, confirming prolonged in vivo stability. Tissue distribution was primarily brain-specific and human TfR1-dependent, with additional enrichment observed in the heart and, potentially, the kidney. with some enrichment in the heart and, potentially, the kidney. Data are presented as mean ± SEM. Statistical analysis was performed using unpaired two-tailed t-test

We selected two heterodimers—TNFI-β-TfR1b-A2 and TNFI-β-TfR1b-D1—for further evaluation of tissue distribution and subcutaneous (SQ) delivery. To test the feasibility of SQ administration—a more patient-friendly alternative to IV, because of its ease of use, steady absorption, reduced clinical resource needs, lower discomfort, and cost efficiency. Approved biologics such as Humira, Enbrel, and Simponi exemplify the success of this route in chronic conditions. In pilot experiments, rats expressing human TfR1 were injected SQ with 1 µL/g of a 40 µM of either TNFI-β-TfR1b-A2 or TNFI-β-TfR1b-D1 solution in PBS. Heterodimers levels were measured 72 hours post-injection. The results are summarized in Fig. 12. In *Tfrc*^*h/w*^ rats, CSF/serum ratios were 0.14 for TNFI-β-TfR1b-A2 and 0.37 for TNFI-β-TfR1b-D1, with average CSF concentrations of 18.8 pM (TNFI-β-TfR1b-A2) and 5.5 pM (TNFI-β-TfR1b-D1). TNFI-β-TfR1b-A2 achieved higher total systemic levels, while TNFI-β-TfR1b-D1 demonstrated superior BBB permeability. Only the heart (and possibly kidney) showed modest signs of tissue distribution dependent on human TfR1 expression. Importantly, heterodimers were detectable in both serum and CSF three days post-injection, indicating markedly extended in vivo stability compared to conventional nanobodies. Furthermore, the consistently higher serum levels in humanized *Tfrc* rats vs. wild-type rats suggest that target-mediated stabilization contributes to this prolonged half-life.

### Assessing in vivo hematotoxicity

**Fig. 13:**
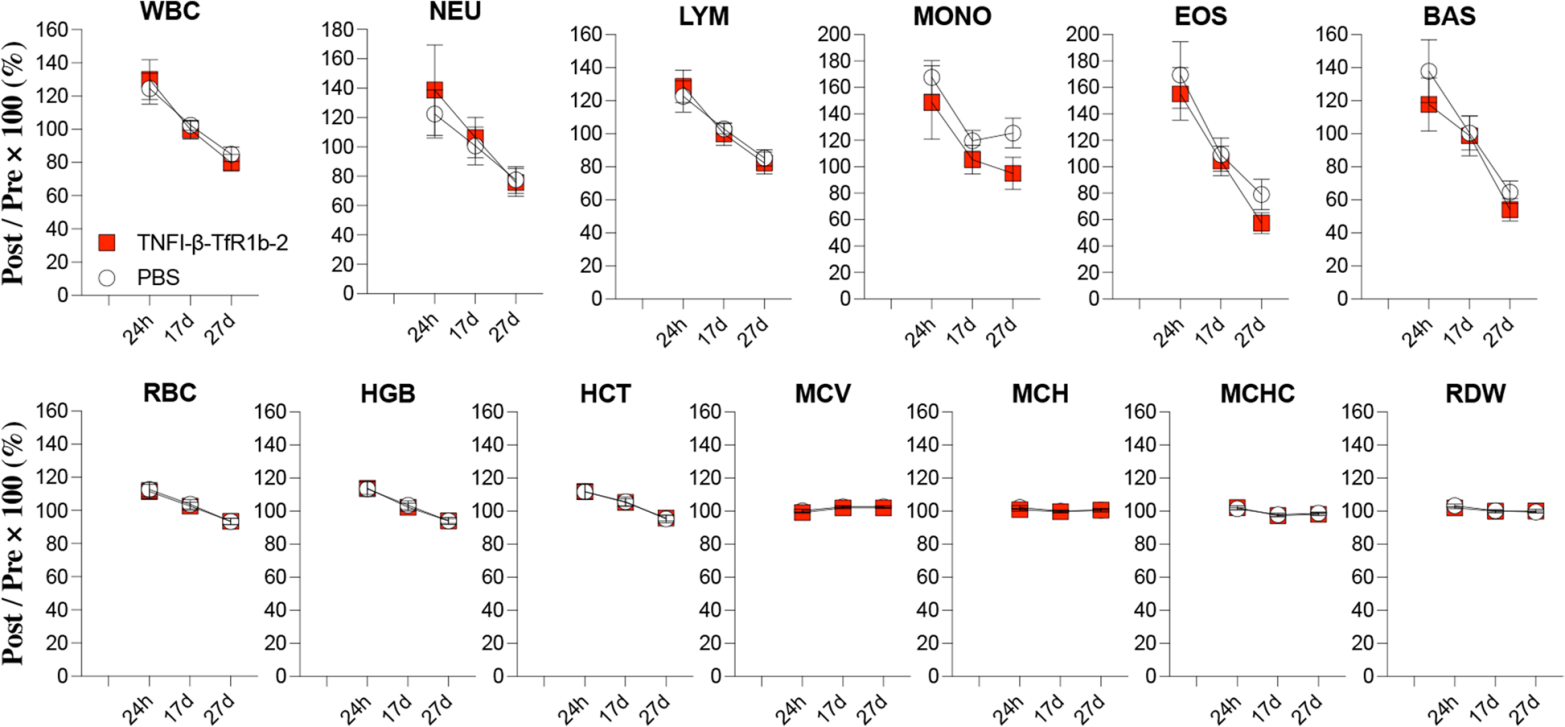
In vivo assessment of hematotoxicity following TNFI-β–TfR1b-A2 administration in humanized rats. In vivo assessment of hematotoxicity following TNFI-β–TfR1b-A2 administration in humanized rats. Complete blood count analysis was performed on ~ 4-month-old rats humanized for both *Tf* and *Tfrc* (*Tf*^*h/w*^*:Tfrc*.^*h/h*^), following IV administration of TNFI-β–TfR1b-A2 or PBS (vehicle control). Post-injection values were normalized to pre-injection baselines and expressed as a percentage (post/pre × 100). “Row” represents time after injection; “column” indicates treatment group. Data are shown as mean ± SEM. Statistical analyses were performed using two-way repeated measures ANOVA (RM ANOVA). A significant main effect of time was observed in all panels except panel MCH. No treatment effects or time × treatment interactions were detected in any panel, and thus, no post hoc comparisons were performed. White Blood Cell Count (WBC): F(1.310, 17.02) = 24.27, *p* < 0.0001 (time); *p* = 0.9102 (treatment); *p* = 0.6059 (interaction). Neutrophils (NEU): F(1.180, 15.34) = 8.548, *p* = 0.0080 (time); *p* = 0.7398 (treatment); *p* = 0.6713 (interaction). Lymphocytes (LYM): F(1.458, 18.95) = 23.61, *p* < 0.0001 (time); *p* = 0.9954 (treatment); *p* = 0.6269 (interaction). Monocytes (MONO): F(1.438, 18.70) = 7.199, p = 0.0087 (time); *p* = 0.1844 (treatment); *p* = 0.7698 (interaction). Eosinophils (EOS): F(1.470, 19.11) = 39.01, *p* < 0.0001 (time); *p* = 0.4711 (treatment); *p* = 0.6727 (interaction). Basophils (BAS): F(1.296, 16.85) = 22.78, *p* < 0.0001 (time); *p* = 0.4394 (treatment); *p* = 0.5806 (interaction). Red Blood Cell Count (RBC): F(1.517, 19.72) = 37.89, *p* < 0.0001 (time); *p* = 0.8441 (treatment); *p* = 0.9000 (interaction). Hemoglobin (HGB): F(1.568, 20.39) = 39.97, *p* < 0.0001 (time); *p* = 0.9378 (treatment); *p* = 0.9000 (interaction). Hematocrit (HCT): F(1.386, 18.01) = 27.87, *p* < 0.0001 (time); *p* = 0.9736 (treatment); *p* = 0.9401 (interaction). Mean Corpuscular Volume(MCV): F(1.338, 17.39) = 22.60, *p* < 0.0001 (time); *p* = 0.2789 (treatment); *p* = 0.7750 (interaction). Mean Corpuscular Hemoglobin (MCH): F(1.302, 16.93) = 2.231, p = 0.1499 (time); p = 0.4857 (treatment); p = 0.5682 (interaction). Mean Corpuscular Hemoglobin Concentration(MCHC): F(1.391, 18.08) = 17.75, *p* = 0.0002 (time); *p* = 0.8376 (treatment); *p* = 0.5267 (interaction). Red Cell Distribution Width (RDW): F(1.121, 14.57) = 12.61, *p* = 0.0024 (time); *p* = 0.7029 (treatment); *p* = 0.2930 (interaction)

Although TfR1b-Nbs from Families D and A do not interfere with TF binding or uptake by CHEK-ATP089 cells (Figs. 2b and 3), we assessed the potential hematotoxicity of heterodimers in vivo. A complete blood count (CBC), conducted by the IRVS core at Rutgers, was performed on ~4 months-old rats humanized for both TF and TfR1, and administered either TNFI-β-TfR1b-A2 or PBS. This is because hematotoxicity, particularly anemia, is potentially influenced by TfR1 binding. The experimental design is detailed in Table 9. The initial CBC (Day −3) established baseline values. Subsequent CBCs (Day 1, Day 17 after three injections, and Day 24 after four injections) monitored for acute and long-term hematotoxic effects. All values remained within normal physiological ranges for rats, indicating that the humanization of *Tf* and *Tfrc* genes has preserved normal blood cell functions. TNFI-β-TfR1b-A2 did not cause significant changes in CBC parameters, including anemia indicators (RBC, HGB, HCT, Fig. 13), compared to PBS controls. Given that these tests were performed in rats expressing human TF and TfR1, the findings are expected to closely reflect the TNFI-β-TfR1b-A2 effects in humans, especially regarding holo-TF-TfR1 interactions and iron uptake. This supports the likelihood of low hematotoxicity in humans, particularly given that the therapeutic dosage of heterodimers may be significantly lower than those tested in these experiments.

**Table 9:** Hematotoxicity assessment. Rats were injected IV on the indicated days (inj.) with either PBS (G1, 3♂/4♀), or TNFI-β-TfR1b-A2 (G2, 3♂/5♀) (1 µL of a 40 µM INN solution in PBS per gram of rat body weight). Complete blood counts (CBC) were performed at several time points: 3 days before the first injection (D −3), 24 hours after the first injection (D1), on Day 17 following three injections, and on Day 24 after four injections. The CBC measurements included white blood cells (WBC), neutrophils (NEU), lymphocytes (LYM), monocytes (MONO), eosinophils (EOS), basophils (BAS), as well as their percentages (NEU %, LYM %, MONO %, EOS %, BAS %), red blood cells (RBC), hemoglobin concentration (HGB), hematocrit (HCT), mean corpuscular volume (MCV), mean corpuscular hemoglobin (MCH), mean corpuscular hemoglobin concentration (MCHC), and red cell distribution width (RDW %)

D −3	D0	D1	D7	D14	D17	D21	D24
CBC	Inj.	CBC	Inj.	Inj.	CBC	Inj.	CBC

In summary, these results demonstrate that heterodimers retain BBB permeability when administered subcutaneously and are viable for human therapeutic use, combining target specificity, extended stability, and ease of delivery with no detectable adverse effects in *Tf-* and *Tfrc*-humanized rat models.

### Surface Plasmon Resonance (SPR) analysis of family A and D NewroBus binding to human TfR1

To determine the binding kinetics and affinity of representative NewroBus nanobodies for human TfR1, SPR analyses were performed using TfR1b-A2 (Family A) and TfR1b-D1 (representative of Family D). Both nanobodies and the recombinant human TfR1 used as ligand were >90% pure, ensuring high-quality reagents for kinetic evaluation.

**Fig. 14:**
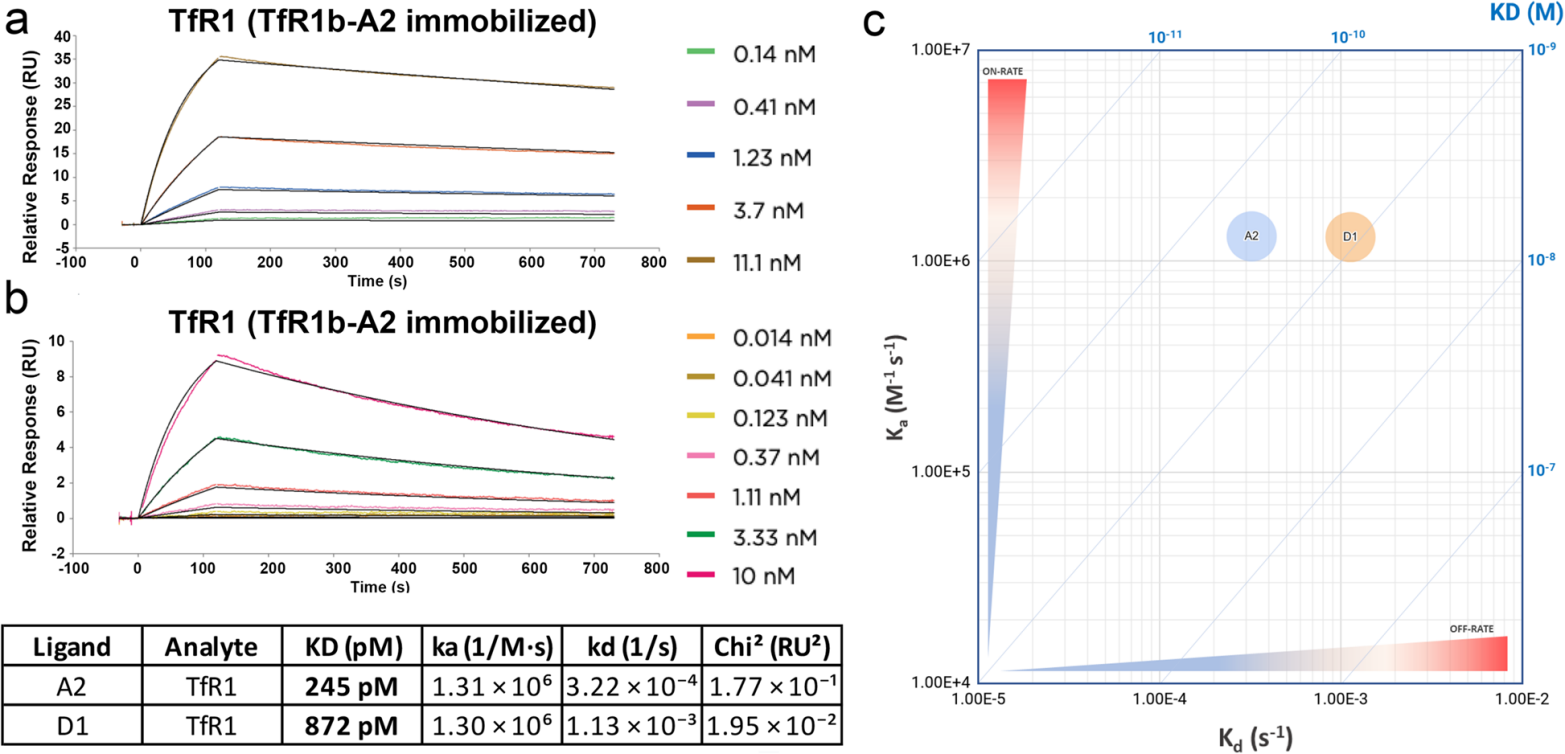
Kinetics of human TfR1 binding to immobilized nanobodies. Kinetic analysis of human TfR1 binding to nanobodies (TfR1b-A2 and TfR1b-D1) immobilized on CMS Carboxyl sensors via amine coupling. **a** Fitted sensorgrams obtained from optimized multi-cycle kinetic analyses of baseline-corrected responses using a 1:1 binding model, with corresponding residuals shown below each fit. Colored lines represent the original sensor traces for five or seven increasing analyte concentrations, prepared as threefold serial dilutions. **b** Summary of kinetic parameters derived from multi-cycle analyses processed with a 1:1 binding model. Goodness of fit was verified by Chi^2^ evaluation and inspection of residual plots. **c** Two-dimensional iso-affinity plot showing association and dissociation rate constants. Blue diagonal lines indicate equilibrium dissociation constants (K_D) and are provided to facilitate visualization of the affinity distribution. Each circle represents rate constant values determined from multi-cycle kinetic analysis using a 1:1 binding model

Multi-cycle kinetic analyses were carried out in both binding orientations—nanobody immobilized as ligand or TfR1 immobilized as ligand—and fitted with a 1:1 Langmuir binding model. In both configurations, A2 and D1 showed specific and dose-dependent interactions with TfR1, exhibiting comparable affinities in the picomolar range. A2 displayed slightly higher affinity than D1 in both orientations, with equilibrium dissociation constants (KD) of 245 pM and 872 pM, respectively, when nanobodies were immobilized as ligands (Fig. 14a and b).

Iso-affinity analysis comparing the association and dissociation rates of A2 and D1 confirmed similar kinetic behavior and binding mechanisms for both nanobodies. These results indicate that both A2 and D1 bind human TfR1 with high affinity and consistent kinetic profiles, validating their selection as strong candidates for BBB transport applications (Fig. 14c).

## Conclusions

This study establishes humanized TfR1b nanobodies (NewroBus) as efficient blood–brain barrier shuttles for CNS-targeted biologics. Lead candidates bind human TfR1 without interfering with transferrin function, show robust brain penetration in humanized TfR1 rats, and exhibit strong developability. Multi-cycle kinetic analyses confirmed high-affinity binding of both A2 and D1 nanobodies to TfR1 in the picomolar range, with A2 displaying the higher affinity (KD = 245 pM vs. 872 pM).

When fused to TNFα-neutralizing nanobodies, NewroBus heterodimers retained BBB permeability and showed extended persistence in serum and CSF following subcutaneous administration—features uncommon for conventional nanobodies. Preliminary data suggest no disruption of iron homeostasis, supporting their favorable safety profile.

**Fig. 15:**
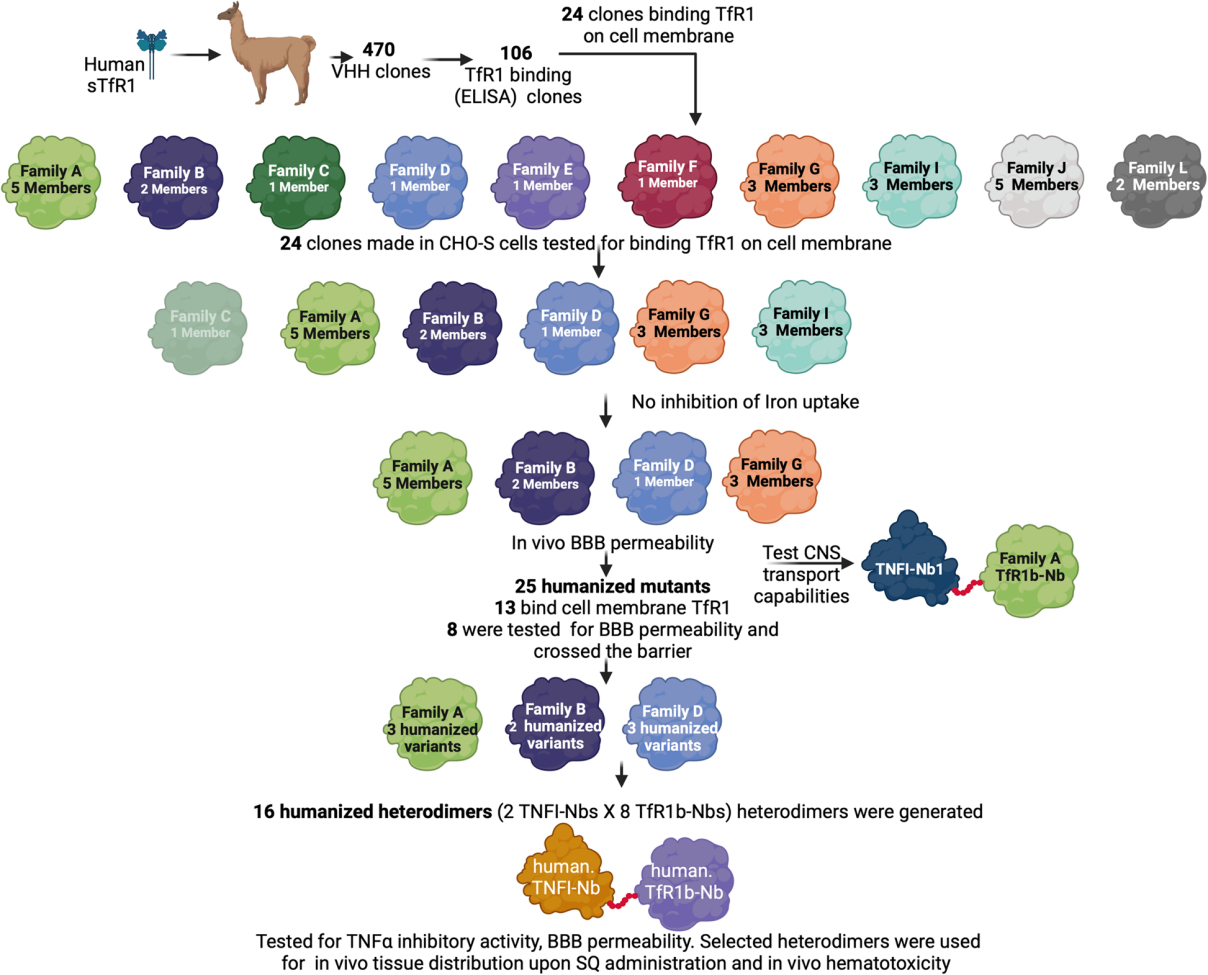
Schematic representation of the workflow for the identification of NewroBus molecules. A step-by-step overview illustrating the process used to discover and optimize NewroBus nanobodies for efficient BBB transcytosis and CNS delivery. The Family C nanobody was not tested

Figure 15 provides a model illustrating how NewroBus hijacks TfR1 on the plasma membrane and appears to undergo transcytosis across the BBB as a neutral passenger, exploiting the natural TfR1–transferrin biological cycle without initiating receptor internalization or competing with holo-Tf.

Future work will refine structure–affinity relationships, assess cross-species reactivity to non-human primate TfR1, and optimize linker architecture to balance CNS selectivity with absolute brain exposure. Collectively, these results position NewroBus as a versatile, human-compatible BBB delivery platform with strong translational potential for chronic CNS therapies.

## Discussion

This study demonstrates the generation and in vivo validation of humanized TfR1b nanobodies, hereafter referred to as NewroBus, as BBB shuttles for CNS-targeted biologics. Key criteria—binding to human TfR1 without disrupting transferrin function, robust CNS penetration in humanized TfR1 rats, and favorable developability profiles—were met by several optimized candidates. Multi-cycle kinetic analyses confirmed high-affinity interactions between the nanobodies and TfR1 in both binding orientations, with A2 and D1 exhibiting picomolar equilibrium dissociation constants (KD = 245 pM and 872 pM, respectively). A2 thus displayed slightly higher affinity, consistent with its strong in vivo performance in CNS delivery.

When fused to TNFα-neutralizing nanobodies, selected NewroBus heterodimers retained BBB permeability and showed extended persistence in serum and CSF after subcutaneous administration, a property not typical of conventional nanobodies. Importantly, preliminary safety data suggest that NewroBus constructs do not impair iron homeostasis, though further studies will be required to confirm tolerability across constructs and doses.

Direct head-to-head comparisons across different TfR1-based platforms are challenging, as published data rely on distinct models, endpoints, and assays, making rankings potentially misleading. Instead, we emphasize the distinguishing features of NewroBus. These nanobodies are fully humanized, bind TfR1 with high affinity and specificity while minimizing interference with transferrin binding and iron homeostasis, and can be modularly fused to therapeutic nanobodies or antibodies. Unlike larger IgG-based shuttles, nanobody constructs may provide advantages in solubility, developability, and design flexibility. Together, these features support their use as versatile brain delivery vehicles.

A modest reduction in BBB delivery efficiency was observed for some heterodimers compared to the corresponding monomeric TfR1 nanobodies. This may reflect the increased molecular size of the constructs, potential changes in binding affinity introduced by fusion, or effects of linker length and composition. Although we did not systematically test these variables here, future studies will be needed to determine how heterodimer design influences BBB permeability.

**Fig. 16:**
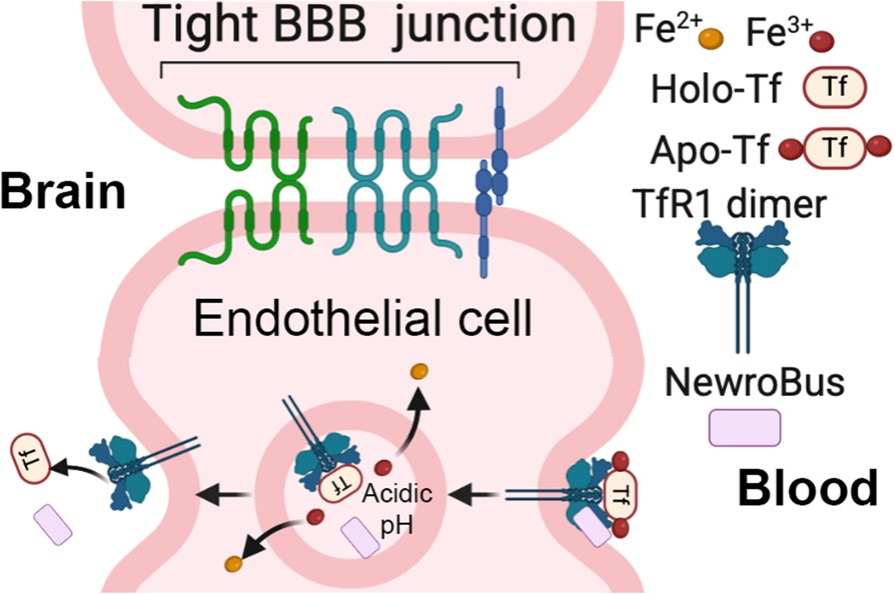
Schematic model of NewroBus hijacking the TfR1–Transferrin pathway to cross the BBB. Holo-transferrin (Fe^3^⁺-loaded transferrin) binds to the dimeric TfR1 at neutral extracellular pH, with two molecules of holo-transferrin engaging each receptor dimer and triggering clathrin-mediated endocytosis. Endosomal acidification induces a conformational change in transferrin that results in Fe^3^⁺ release, followed by transport of Fe^2^⁺ into the cytosol. Apo-transferrin remains bound to TfR1 during trafficking back to the cell surface, where neutral pH promotes dissociation. NewroBus engages a distinct non-competitive epitope spatially separate from the transferrin-binding region and does not trigger TfR1 internalization because of his monomeric nature, thereby avoiding interference with iron uptake*.* Instead, NewroBus is transcytosed across endothelial cells of the BBB by leveraging the natural TfR1 recycling pathway and is released at least in part within acidified endosomes and/or during receptor recycling to the blood-facing membrane surface

In summary, we generated and optimized NewroBus constructs that cross the BBB in a TfR1-dependent manner without disrupting transferrin function. When fused to TNFα-neutralizing nanobodies, they preserved BBB permeability, maintained target engagement, and displayed prolonged persistence in CSF and serum after subcutaneous delivery. Preliminary safety data suggest minimal risk of iron homeostasis disruption, though broader studies are needed. Together, these results position NewroBus as a versatile BBB shuttle with strong translational potential for chronic CNS therapy, and the overall pipeline—from immunization to in vivo validation and humanization—is summarized in Fig. 16.

### Limitations and future directions

While this study establishes humanized TfR1b-Nbs (NewroBus molecules) as highly promising BBB shuttles for therapeutic biologics, several important limitations remain and warrant further investigation.

First, structural studies—such as cryo-electron microscopy (cryo-EM)—would provide valuable insight into how TfR1b-Nbs engage their target at the molecular level. Structural resolution of nanobody–TfR1 complexes could identify precise epitopes, confirm whether the nanobodies bind to one or both subunits of the TfR1 dimer, and whether their binding site is distinct from the transferrin binding site. This information would support the rational design of next-generation molecules that retain TfR1 function while maximizing receptor engagement and transcytosis efficiency.

Second, the cross-reactivity of TfR1b-Nbs with non-human primate (NHP) TfR1 remains to be evaluated. Assessing binding to monkey TfR1 is critical to determine the suitability of NHP models for preclinical safety, pharmacokinetic, and toxicology studies. If cross-reactivity is lacking, alternative strategies may include the development of NHP-compatible surrogate nanobodies, or the continued use of rodent models humanized for TfR1 and transferrin.

All tested nanobody constructs, including heterodimers, demonstrated favorable CSF/serum ratios (>0.1), indicative of BBB transcytosis. However, key differences were observed, and a critical question remains: What is the optimal balance between brain selectivity and absolute CNS exposure? Some constructs exhibited high CSF/serum ratios (~1.0) but relatively lower overall nanobody concentrations in both compartments, while others showed higher absolute levels with more moderate CSF/serum ratios (<0.5). A high CSF/serum ratio may reduce the risk of systemic side effects by limiting peripheral target engagement, whereas high absolute CNS concentrations may be more therapeutically advantageous in diseases requiring potent inhibition of central inflammatory pathways, such as TNFα-driven neuroinflammation.

Furthermore, the relevance of peripheral TNFα inhibition is likely disease-specific. In cases where central inflammation is accompanied by systemic inflammatory components—or where peripheral TNFα contributes to CNS pathology—some degree of peripheral TNFα blockade may be therapeutically beneficial.

In summary, future work will focus on defining nanobody–TfR1 binding affinities, assessing cross-species reactivity, solving nanobody–TfR1 structures, and refining pharmacokinetic and pharmacodynamic profiles. These studies will enable the rational optimization of NewroBus molecules for specific CNS indications and accelerate their progression toward clinical application.

## Conclusion

Taken together, our findings demonstrate that humanized TfR1b-Nbs enable efficient, TfR1-dependent transcytosis across the BBB, delivering otherwise impermeable therapeutic payloads into the CNS. These nanobodies exhibit favorable pharmacokinetics, minimal hematotoxicity, and sustained CNS exposure following subcutaneous administration. Moreover, their ability to localize to astrocytes and microglia highlights their potential for cell-targeted intervention in neuroinflammatory and neurodegenerative diseases. These TfR1b-Nbs have been exclusively licensed to NanoNewron and are collectively referred to as NewroBus molecules. As such, NewroBus represents a promising platform for advancing the delivery of biologics to the brain.

## Supplementary Information

Supplementary Material 1.

## Data Availability

No datasets were generated or analysed during the current study.
