# Supplementary material for: The NewroBus platform: engineered humanized anti-TfR1 nanobodies for efficient brain delivery
Source: Cell Commun Signal. 2025 Dec 30;24:69. doi: 10.1186/s12964-025-02605-1 (PMC12853688; doi:10.1186/s12964-025-02605-1)
Supplement: Supplementary file 1 — Supplementary Material 1. [file 12964_2025_2605_MOESM1_ESM.docx]

**Supplemental Figures**

**
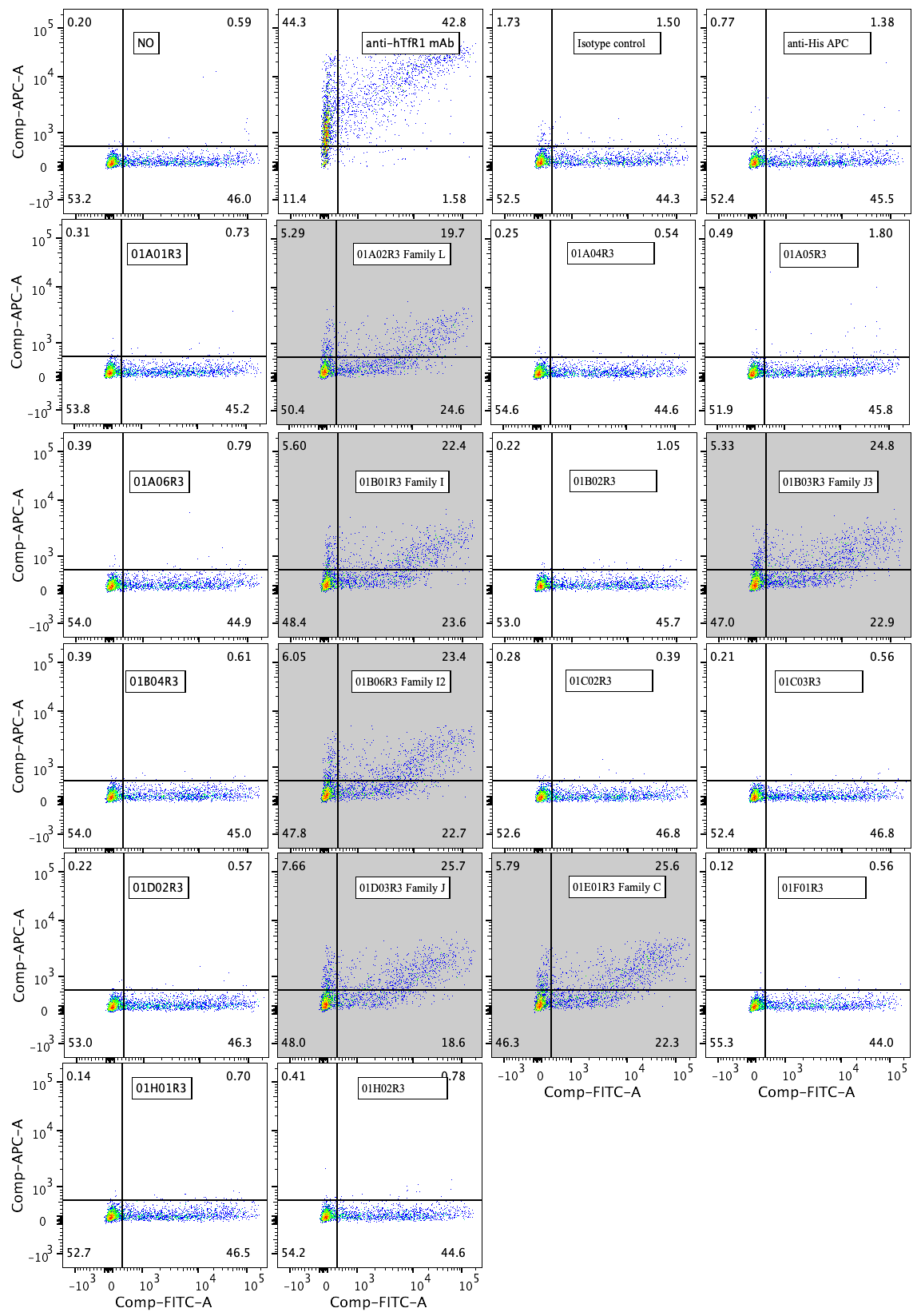
**

**Figure S1. Screening of 106 bacterially produced anti-TfR1 nanobodies for binding to cell-surface human TfR1**. The first four panels show control staining: No (unstained, negative control); anti-hTfR1–APC antibody (positive control); isotype control–APC (negative control); and anti-His–APC (negative control, secondary antibody used to detect nanobodies). These controls confirm that transfection specifically induces expression of human TfR1 and EGFP. The remaining panels show staining results for the first 22 anti-TfR1 nanobodies tested for binding to human TfR1. The clones binding human TfR1 are in grey.


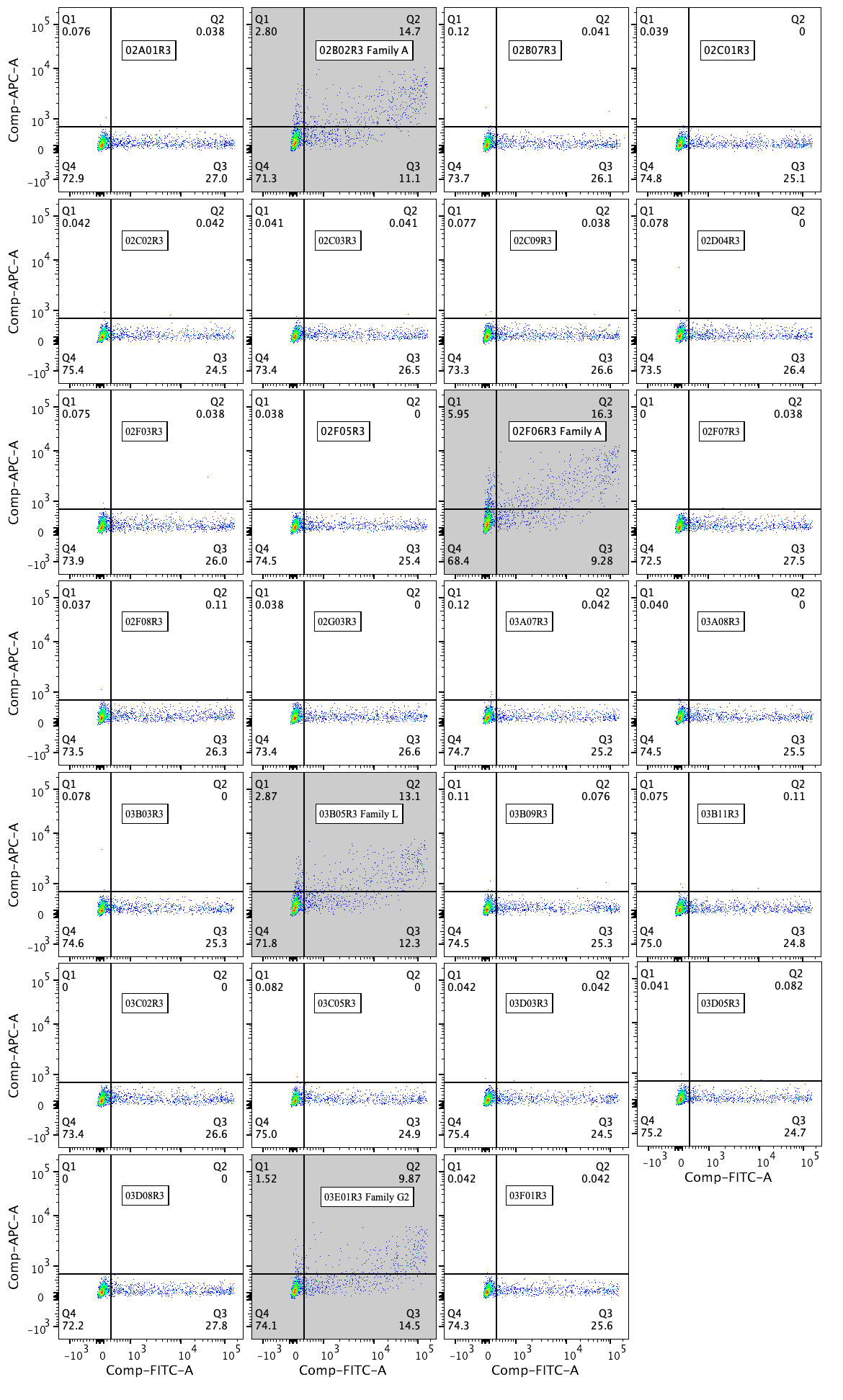


**Figure S2. Screening of 106 bacterially produced anti-TfR1 nanobodies for binding to cell-surface human TfR1**. Staining results of 27 more anti-TfR1 nanobodies tested for binding to human TfR1. The clones binding human TfR1 are in grey.


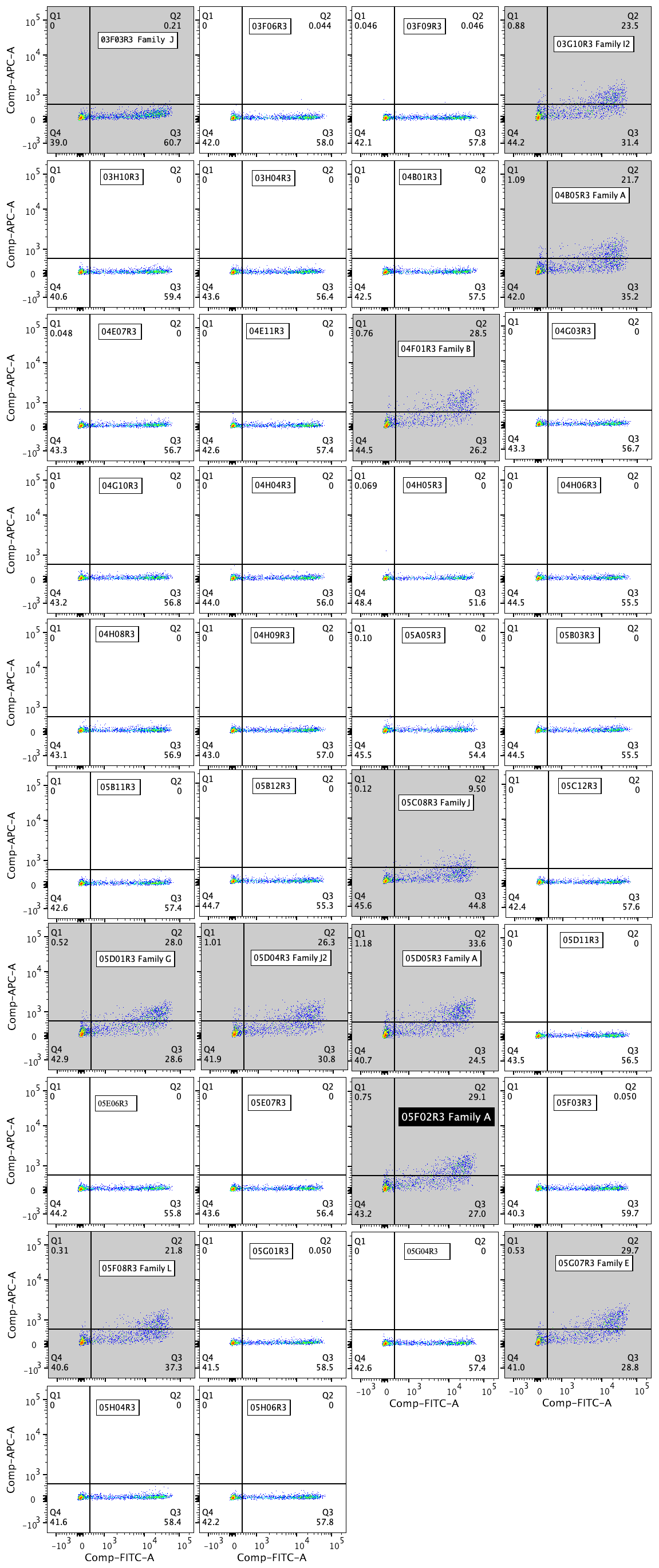


**Figure S3. Screening of 106 bacterially produced anti-TfR1 nanobodies for binding to cell-surface human TfR1**. Staining results of 38 more anti-TfR1 nanobodies tested for binding to human TfR1. The clones binding human TfR1 are in grey. The Nb highlighted in black is one of the two parental sequences from which the final selected NewroBus molecules were derived.

**
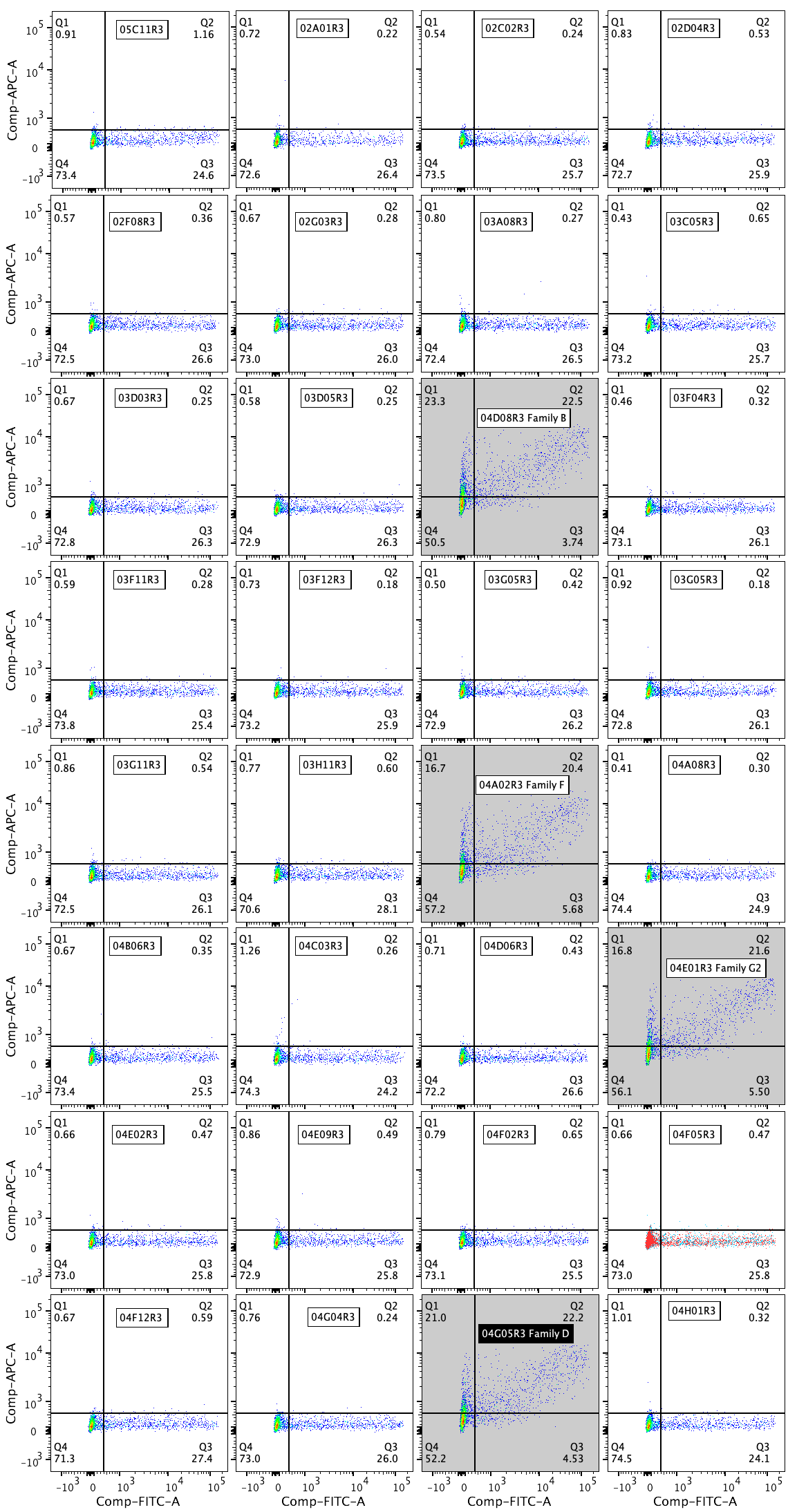
**

**Figure S4. Screening of 106 bacterially produced anti-TfR1 nanobodies for binding to cell-surface human TfR1**. Staining results of 32 more anti-TfR1 nanobodies tested for binding to human TfR1. The clones binding human TfR1 are in grey. The Nb highlighted in black is one of the two parental sequences from which the final selected NewroBus molecules were derived.

**
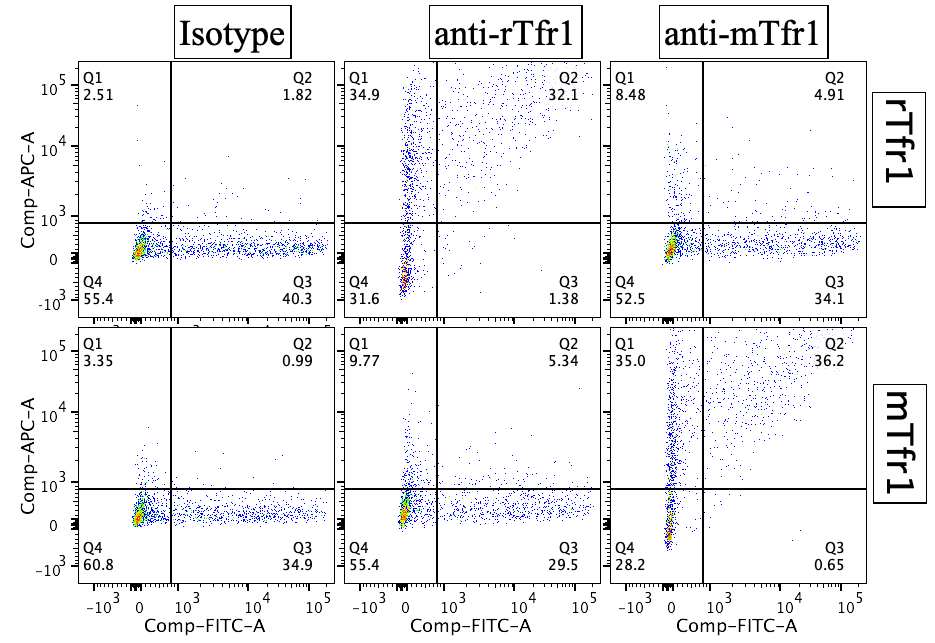
**

**Figure S5. Transfection of plasmids expressing mouse or rat TfR1 to assess antibody specificity.** HEK293 cells were transfected with plasmids encoding either mouse Tfr1 or rat Tfr1. Specificity of detection was assessed using an isotype control, an anti-mouse Tfr1–APC antibody, and an anti-rat Tfr1–APC antibody. The anti-mouse Tfr1 antibody selectively stained cells transfected with mouse Tfr1, while the anti-rat Tfr1 antibody selectively stained cells expressing rat Tfr1, confirming species-specific binding of each antibody.

**
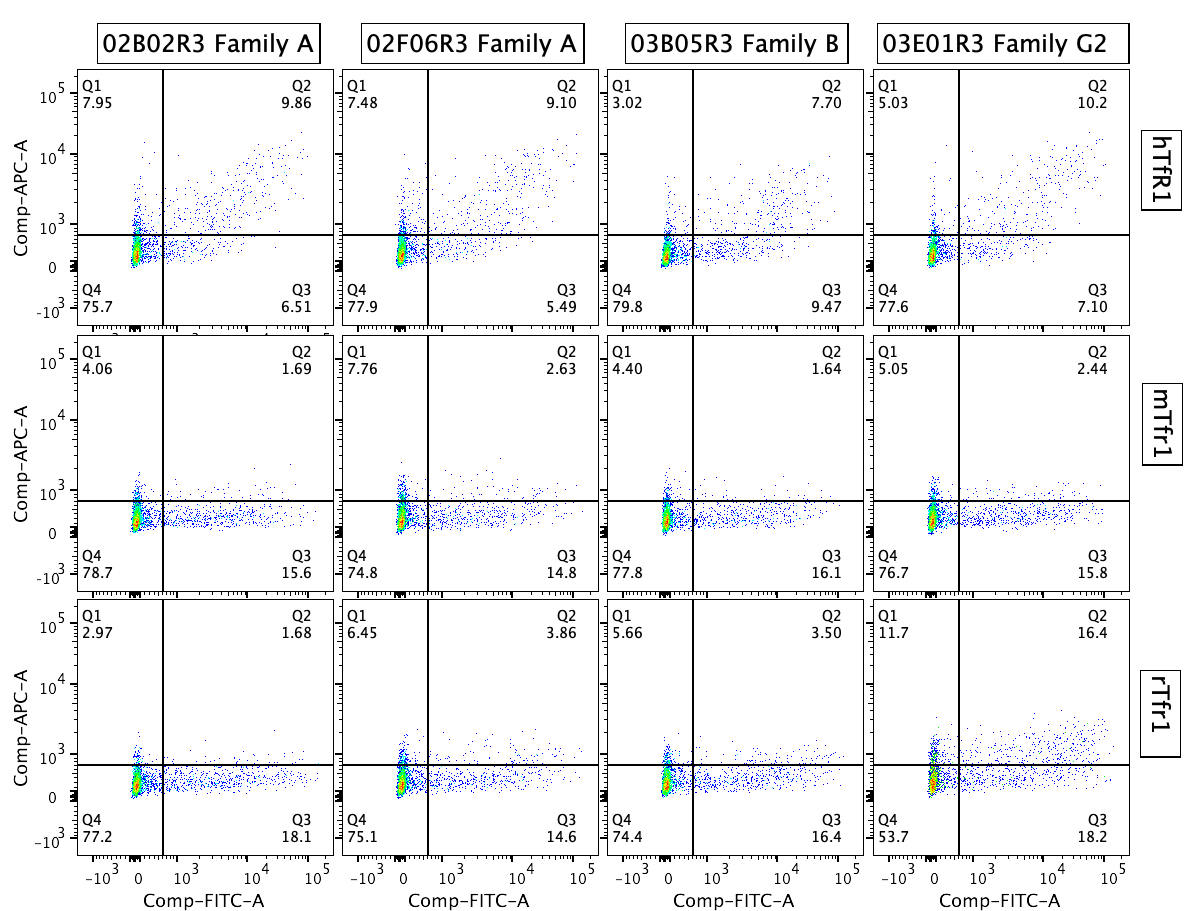
**

**Figure S6. Part 1: Tfr1b-Nbs recognize human TfR1 but not rat and mouse Tfr1.**


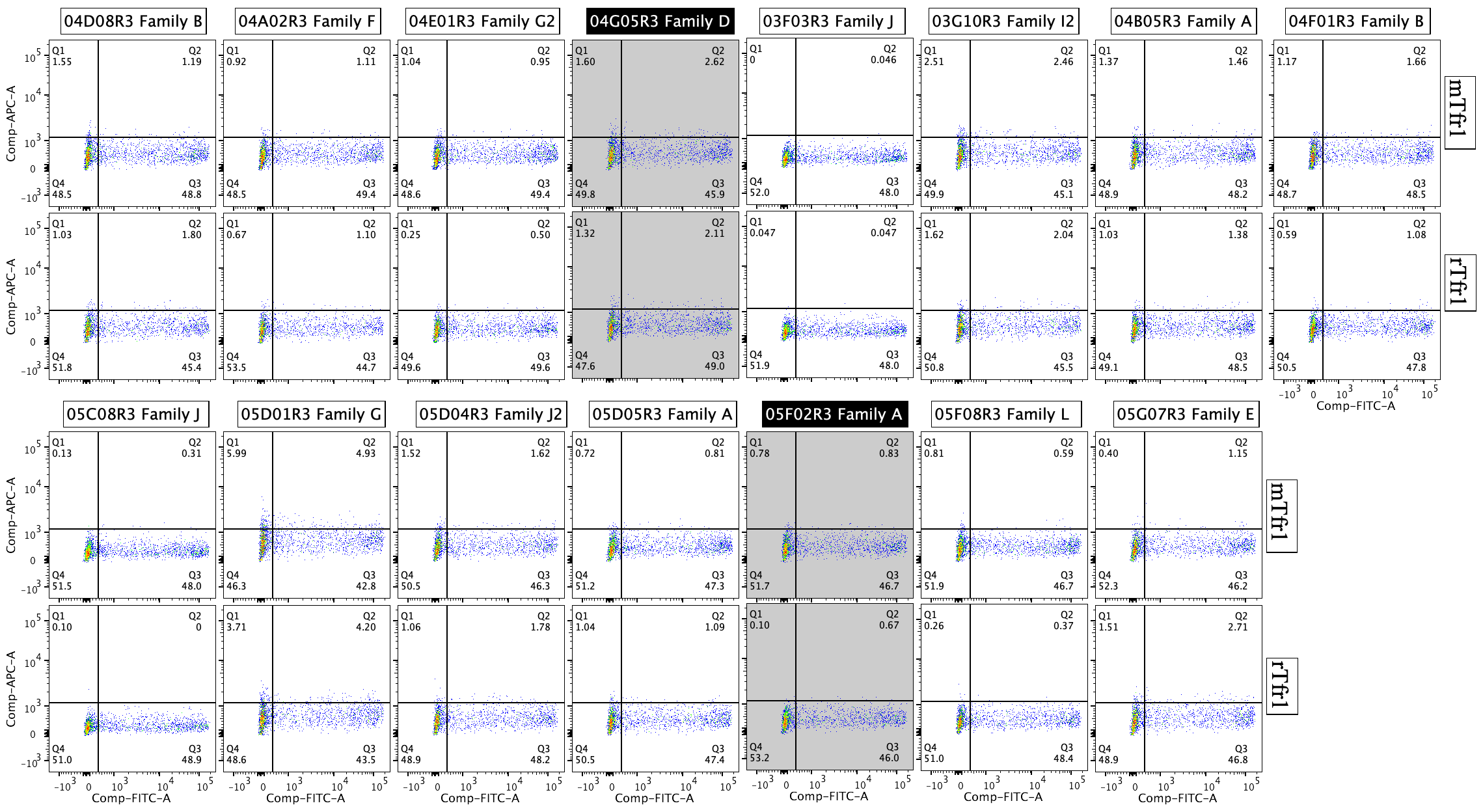


**Figure S7. Part 2: Tfr1b-Nbs do not recognize rat and mouse Tfr1.** The Nbs highlighted in black are the parental sequences from which the final selected NewroBus molecules were derived.


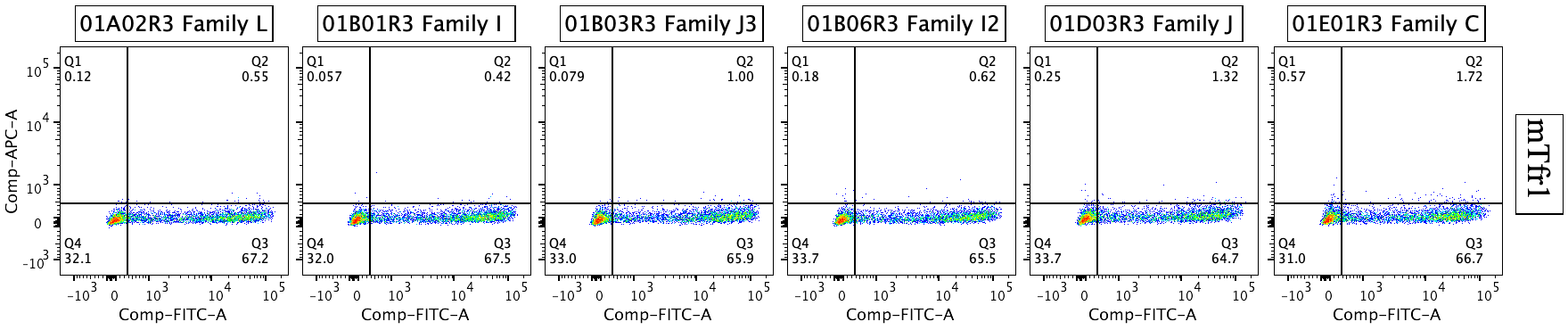


**Figure S8. Part 3: Tfr1b-Nbs do not recognize mouse Tfr1.**


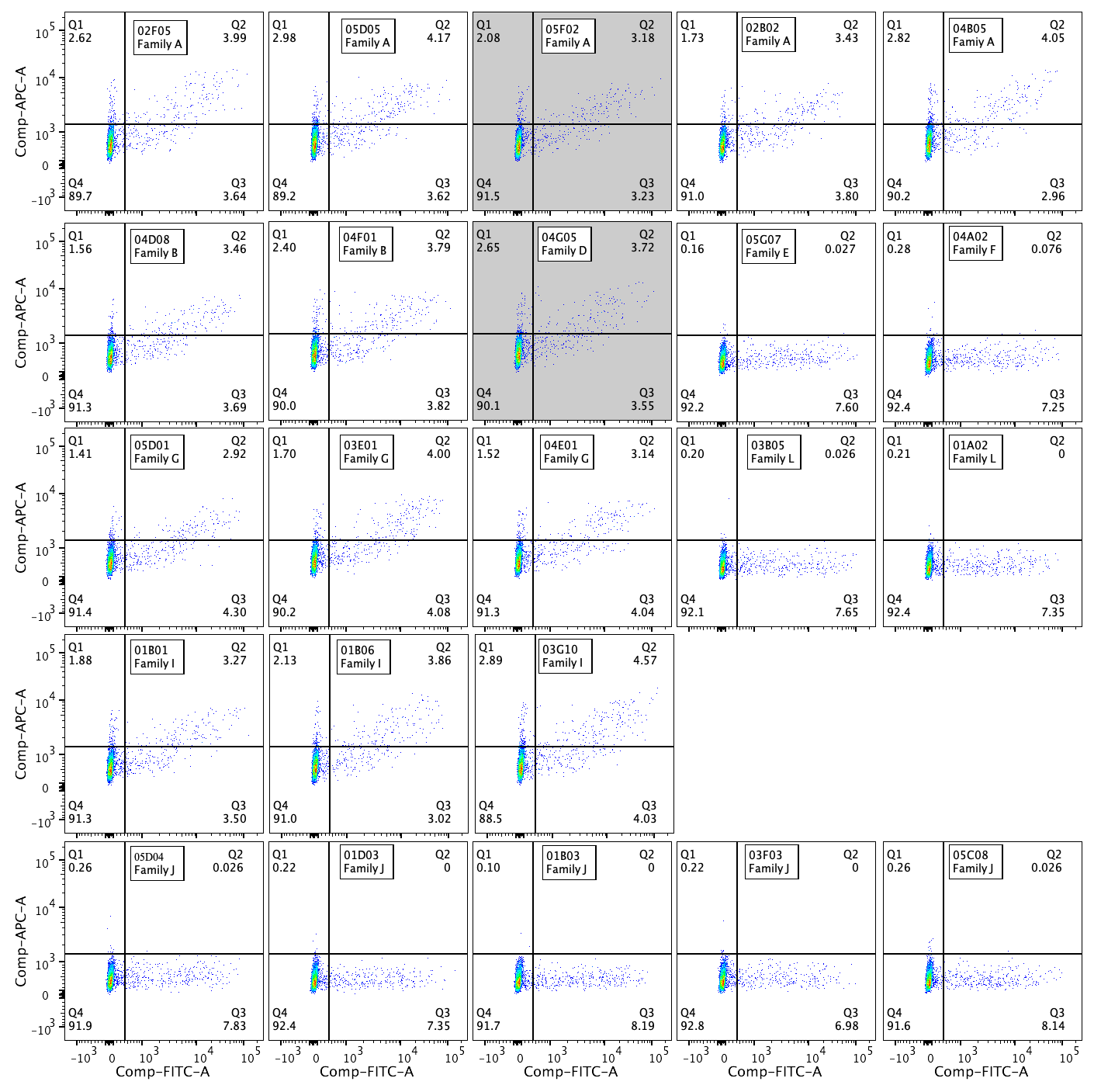


**Figure S9. TfR1b-Nbs produced by mammalian cells bind to human TfR1.** HEK293 cells were transfected with a vector expressing human TfR1 (alongside EGFP). Subsequently, cells were treated with each TfR1b-Nb at a concentration of 400 nanomolar, followed by incubation with an anti-His-APC antibody. The two nanobodies highlighted in grey are the parental sequences from which the final selected NewroBus molecules were derived.
